# SPOP and HAUSP bidirectionally regulate LZTS2 ubiquitination to modulate the Wnt pathway

**DOI:** 10.1038/s41419-025-08351-z

**Published:** 2025-12-24

**Authors:** Yanran Deng, Chunfan Xie, Ran Liu, Kaize Ma, Jian Tang, Zizhang Zhou

**Affiliations:** 1 Key Laboratory of Biodiversity Conservation and Bioresource Utilization of Jiangxi Province, College of Life Sciences, Jiangxi Normal University, Nanchang, China; 2 Department of Thoracic Surgery, Nanfang Hospital, Southern Medical University, Guangzhou, China; 3 Department of Thoracic Surgery, The First Affiliated Hospital, Jiangxi Medical College, Nanchang University, Nanchang, China; 4 College of Life Sciences, Shandong Agricultural University, Tai’an, China

**Keywords:** Colon cancer, Tumour-suppressor proteins

## Abstract

Colorectal cancer (CRC) is a highly deadly disease worldwide, often characterized by the overactivation of the Wnt pathway. LZTS2 is known to be a tumor suppressor by negatively regulating the Wnt pathway in CRC. However, the mechanisms that control the stability of LZTS2 are not fully understood. In this study, we find that the E3 ligase SPOP promotes ubiquitination-mediated degradation of LZTS2, which is counteracted by the deubiquitinase HAUSP. SPOP and HAUSP compete for binding to the same region of LZTS2, leading to bidirectional regulation of LZTS2 stability. The regulation ultimately impacts the activity of the Wnt pathway. Furthermore, functional analyses reveal that SPOP hinders the tumor-suppressive effects of LZTS2 on CRC cell proliferation and metastasis, whereas HAUSP enhances LZTS2’s anti-tumor activity in CRC cells. Taken together, these findings uncover a novel regulatory mechanism of LZTS2 stability, where SPOP and HAUSP play crucial roles in determining the behavior of CRC cells by balancing the ubiquitination and deubiquitination of LZTS2. This discovery may offer new strategies for utilizing LZTS2 as a potential therapeutic target for cancer treatment.

## Introduction

Colorectal cancer (CRC) is among the most lethal and prevalent malignancies globally and leads to about one million cancer-related deaths every year [1]. Traditional treatment options for CRC patients include surgery, chemotherapy and radiation, but they face challenges such as cancer recurrence, toxicity and high side effects [2]. The development of targeted therapy has shown promising results in improving treatment outcomes for CRC patients [3]. However, drug resistance and the lack of suitable targeted drugs remain major obstacles in effective treatment [4]. Therefore, there is an urgent need to investigate the molecular mechanisms of CRC progression and identify new targets for clinical treatment.

Overactivation of the Wnt pathway is one of the important mechanisms for the initiation and progression of CRC [5]. Wnt serves as a ligand that binds to the transmembrane receptor complex composed of Frizzled (FZD) and low-density lipoprotein receptor-related protein (LRP), leading to signaling activation [6, 7]. In the absence of the Wnt ligand, the transcription factor β-catenin is tethered in the cytoplasm by a destruction complex, containing AXIN, APC, GSK3β and CK1α^7^. In this state, β-catenin is phosphorylated, ubiquitinated by β-TrCP and consequently degraded through proteasome [8]. Without nuclear β-catenin, a repressive complex with TCF/LEF and TLE/Groucho recruits HDACs to suppress the expression of target genes [9]. Upon binding of Wnt ligand to the FZD/LRP receptor, β-catenin is released from the destruction complex and enters the nucleus to destroy the repressive complex, thereby activating target genes [10]. Therefore, nuclear translocation of β-catenin is essential for Wnt pathway activation. Inhibiting β-catenin nuclear localization could be a potential strategy to block the Wnt pathway and treat CRC. In fact, several inhibitors of β-catenin nuclear localization are being investigated for their possible anti-tumor effects [11–13].

LZTS2, a leucine zipper tumor suppressor 2, is originally discovered as a homolog to LZTS1 [14]. *LZTS2* gene is located on human chromosome 10q24.3, a region frequently deleted in various cancers, including prostate tumor and CRC [14]. Studies have shown that ectopic expression of LZTS2 apparently inhibited tumor cell proliferation, suggesting its role as a tumor suppressor [14]. A yeast two-hybrid screen using human β-catenin as bait identifies LZTS2 as an interacting partner [15]. In CRC, LZTS2 interacts with β-catenin to diminish its nuclear localization, thereby inhibiting the activation of the Wnt pathway and tumorigenesis [15]. Our recent study has revealed that the oncogenic kinase PLK1 phosphorylates LZTS2 to decrease its interaction with β-catenin, leading to Wnt pathway activation and progression of lung adenocarcinoma [16]. LZTS2 is able to collaborate with PTEN in suppressing prostate tumorigenesis by reducing β-catenin-mediated transcription [17]. Additionally, LZTS2 inhibits tumorigenesis and radioresistance in nasopharyngeal carcinoma through inhibiting the PI3K/AKT pathway [18]. Therefore, LZTS2 may employ different mechanisms for tumor suppression depending on the context. Interestingly, *LZTS2* has been identified as an oncogene and an independent prognostic biomarker for clear cell renal cell carcinoma [19], indicating its complex roles in tumorigenesis. While many studies are focusing on the downstream effects of LZTS2, the regulation of its own abundance remains unclear.

The ubiquitin-proteasome system (UPS) is a vital pathway for the degradation of intracellular proteins, controlled by the opposing actions of E3 ubiquitin ligases and deubiquitinating enzymes (DUBs) [20]. Maintaining a balance between ubiquitination and deubiquitination is essential for proper protein levels, as any disruption can lead to various diseases, including cancer [21]. The speckle-type pox virus and zinc finger (POZ) protein (SPOP) is a well-known adaptor for CUL3-based E3 ligase complex, determining which proteins are targeted for ubiquitination and degradation [22, 23]. Structurally, SPOP consists of a MATH domain for substrate recognition and a BTB domain for binding CUL3 [22]. Increasing studies have shown that most of SPOP substrates are oncoproteins [23–28], indicating its anti-tumor roles. However, our previous study has revealed that SPOP can also target the tumor suppressor IRF2BP2, leading to progression of liver cancer [29]. After analyzing the mass spectrometry results using SPOP as bait from our study [29] and another group [30], it was found that LZTS2 could be a potential interacting partner of SPOP. We carried out rigorous biochemical experiments and confirmed that LZTS2 was a bona fide substrate of CUL3-SPOP E3 ligase. In addition, previous unbiased tandem affinity purification and mass spectrometry studies have pinpointed HAUSP (also known as USP7) as the most probable LZTS2-interacting protein, ranking at the top of all potential candidates [18]. This suggests that HAUSP may have the ability to reverse LZTS2 ubiquitination. Nevertheless, it is still unclear whether LZTS2 is subject to bidirectional regulation by both SPOP and HAUSP, as well as the biological significance of this regulation.

In this study, we provide biochemical evidence to support that LZTS2 is a substrate of both SPOP and HAUSP. SPOP binds to LZTS2 through its N-terminal MATH domain and facilitates LZTS2 ubiquitination in a CUL3-dependent manner. HAUSP competes with SPOP for binding to the same region of LZTS2, leading to deubiquitination of LZTS2. Furthermore, LZTS2 restricts β-catenin in the cytoplasm and diminishes its transcriptional activity, which is counteracted by SPOP and exacerbated by HAUSP. Functionally, LZTS2 suppresses the proliferation and migration of CRC cells, with these effects being attenuated by co-expression of SPOP but strengthened by co-expressing HAUSP. Overall, these findings not only uncover a bidirectional regulatory mechanism of LZTS2 abundance but also demonstrate that SPOP and HAUSP regulate the Wnt pathway by modulating LZTS2.

## Results

### LZTS2 is a binding protein of SPOP

**Fig. 1:**
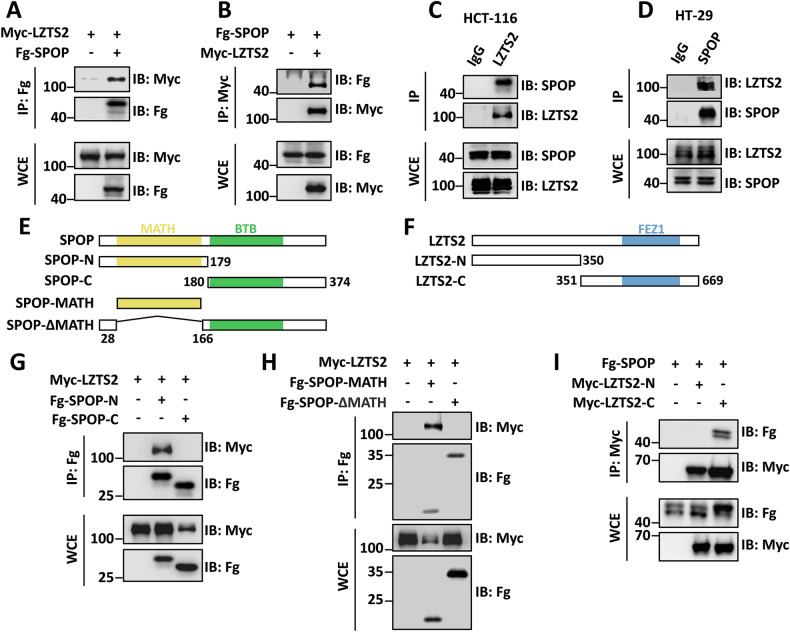
SPOP interacts with the C-terminus of LZTS2 via its MATH domain. **A** Immunoblots of immunoprecipitates (IP, top two panels) or whole cell extracts (WCE, bottom two panels) from 293 T cells expressing indicated plasmids and treated with 25 μM of MG132 for 4 h before cell harvesting. Of note, Fg-SPOP could pull down Myc-LZTS2. **B** Myc-LZTS2 was able to pull down Fg-SPOP. **C** Endogenous LZTS2 interacted with endogenous SPOP in HCT-116 cells. **D** Endogenous SPOP immunoprecipitated endogenous LZTS2 in HT-29 cells. Schematic drawings display the domains in SPOP (**E**) and LZTS2 (**F**), along with the truncated mutants utilized in the subsequent studies. **G** Fg-SPOP-N could, but Fg-SPOP-C could not immunoprecipitate Myc-LZTS2. **H** Fg-SPOP-MATH, but not Fg-SPOP-ΔMATH interacted with Myc-LZTS2 in 293 T cells. **I** Fg-SPOP interacted with Myc-LZTS2-C, not Myc-LZTS2-N.

Increasing studies have highlighted the important role of LZTS2 in inhibiting tumorigenesis in various types of cancer, including CRC. Understanding the regulatory mechanism of LZTS2 could provide new therapeutic opportunities for CRC patients. In our previous study, a coimmunoprecipitation coupled with mass spectrometry (Co-IP/MS) approach was used to identify proteins that interact with the E3 ligase SPOP in HEK-293T (293 T) cells, leading to the discovery of LZTS2 as a potential interacting partner [29]. To validate this finding, we expressed Fg-tagged SPOP and Myc-tagged LZTS2 constructs in 293 T cells and carried out Co-IP experiments. The results revealed that Fg-SPOP binds to Myc-LZTS2 (Fig. 1A). Reciprocally, Myc-LZTS2 also pulled down Fg-SPOP (Fig. 1B). In addition, the interaction between endogenous SPOP and endogenous LZTS2 was observed in both HCT-116 (Fig. 1C) and HT-29 cells (Fig. 1D), indicating that LZTS2 is a genuine interacting partner of SPOP in CRC cells.

In order to determine the domains responsible for the interaction between SPOP and LZTS2, we generated truncated forms of both proteins for Co-IP assays (Fig. 1E-F). SPOP contains a N-terminal MATH domain for substrate binding, and a C-terminal BTB domain for recruiting CUL3 and self-oligomerization [22, 31]. We divided SPOP into SPOP-N and SPOP-C (Fig. 1E), and found that only SPOP-N was able to pull down LZTS2, while SPOP-C was not (Fig. 1G). Additionally, we constructed Fg-SPOP-MATH and Fg-SPOP-ΔMATH plasmids to investigate the role of the MATH domain for SPOP-LZTS2 interaction (Fig. 1E). It was revealed that SPOP-MATH, but not SPOP-ΔMATH, interacted with LZTS2 (Fig. 1H), demonstrating a MATH-dependent manner for SPOP to bind LZTS2. On the other hand, we revealed that the C-terminus of LZTS2, rather than the N-terminus was responsible for its interaction with SPOP (Fig. 1I). These findings collectively reveal that SPOP binds the C-terminus of LZTS2 through its MATH domain.

### SPOP promotes the ubiquitination and degradation of LZTS2

**Fig. 2:**
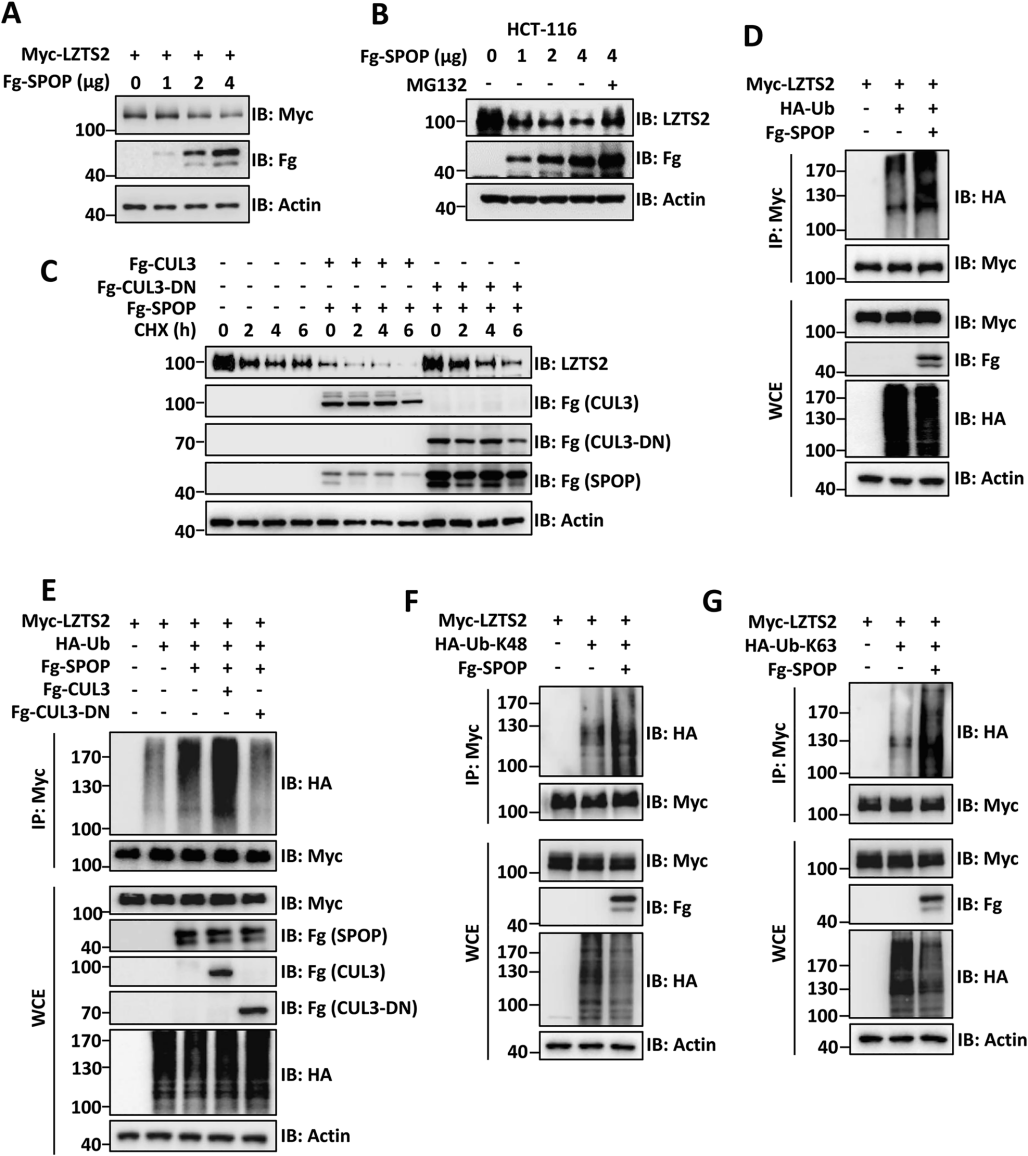
SPOP ubiquitinates and degrades LZTS2. **A** SPOP decreased Myc-LZTS2 protein in a dose-dependent manner in 293 T cells. **B** SPOP degraded endogenous LZTS2 in a dose-dependent manner in HCT-116 cells, which was restored by treatment with 25 μM of proteasome inhibitor MG132 for 4 h. **C** 293 T cells were treated with CHX (20 μg/ml) to inhibit the protein synthesis for indicated times before harvesting. Of note, CUL3 promoted, while CUL3-DN inhibited SPOP-mediated LZTS2 degradation. **D** Immunoblots of immunoprecipitates (top two panels) or whole cell extracts (bottom three panels) from 293 T cells transfected with the indicated plasmids and treated with 25 μM of MG132 for 6 h before cell harvesting. SPOP enhanced ubiquitination of LZTS2. **E** CUL3 enhanced, while CUL3-DN inhibited SPOP-mediated LZTS2 ubiquitination. **F** SPOP promoted K48-linked polyubiquitination on LZTS2. **G** SPOP facilitated K63-linked polyubiquitination on LZTS2. Above all, Actin acts as a loading control.

The discovery that SPOP binds to LZTS2 through its MATH domain, promoted us to investigate whether LZTS2 is a target for ubiquitination by SPOP. To explore this, we introduced Myc-LZTS2 and increasing amounts of Fg-SPOP into 293 T cells. Western blot analysis revealed that Fg-SPOP decreased the protein levels of Myc-LZTS2 in a dose-dependent manner (Figs. 2A and S2A). In HCT-116 cells, Fg-SPOP was able to reduce endogenous LZTS2, which was reversed by treatment with the proteasome inhibitor MG132 (Figs. 2B and S2B). Nevertheless, the degradation of Myc-LZTS2 protein by Fg-SPOP was not affected by the lysosomal inhibitor NH_4_Cl (Fig. S1A), indicating that SPOP facilitates LZTS2 destabilization through the proteasome. It is known that SPOP requires the scaffold CUL3 to carry out its E3 ligase function [32]. The C-terminal of CUL3 associates with the RING-domain proteins RBX1 or RBX2, which facilitate the transfer of Ubiquitin (Ub) from the Ub-conjugating enzymes (E2s) to the substrates [33]. Through its N-terminal domain, CUL3 interacts with specific adaptor-substrate components [34]. Therefore, we tested whether SPOP-mediated degradation of LZTS2 is dependent on CUL3. Notably, LZTS2 protein displayed instability with a half-life of ~2 h in the presence of the translation inhibitor cycloheximide (CHX) (Figs. 2C and S2C). This half-life was shortened when both CUL3 and SPOP were present (Figs. 2C and S2C). We created a truncated version of CUL3, CUL3-aa1-595, which lacks the C-terminus and cannot recruit E2s. This truncated version was labeled as CUL3-DN because of its dominant-negative role, as it competes with endogenous CUL3 for adaptor binding [35, 36]. When CUL3-DN was co-transfected with SPOP, it successfully halted the degradation of LZTS2 (Figs. 2C and S2C), suggesting that SPOP targets LZTS2 for degradation via the SPOP-CUL3 complex.

After demonstrating that SPOP-CUL3 promotes the degradation of LZTS2, we proceeded to investigate the ubiquitination of LZTS2. As expected, SPOP indeed enhanced the ubiquitination of LZTS2 both in vivo and in vitro (Figs. 2D and S1B). Furthermore, this ubiquitination was strengthened by wild-type CUL3, but weakened by CUL3-DN (Fig. 2E), underscoring the crucial role of CUL3 in SPOP-induced ubiquitination of LZTS2. As SPOP-mediated ubiquitination of LZTS2 leads to degradation, we then examined the type of polyubiquitination chain on LZTS2. Since K48-polyUb-modified proteins are typically targeted for degradation, we utilized Ub-K48, where the lysines (Ks) on Ub are substituted with arginines except for K48. Co-IP results showed that SPOP promoted K48-linked ubiquitination on LZTS2 (Fig. 2F). While various types of polyubiquitin chains exist, K48- and K63-linked polyubiquitination are the most common types of linkage [37]. Interestingly, Ub-K63 exhibited a similar effect to Ub-K48 on SPOP-mediated LZTS2 ubiquitination (Fig. 2G). We also tested other types of polyubiquitin chains. However, SPOP failed to ubiquitinate LZTS2 in the presence of K6-, K11-, K27-, K29-, and K33-linked ubiquitin chains (Fig. S1C-G). Taken together, our findings indicate that SPOP recruits CUL3 to catalyze K48-linked polyubiquitination on LZTS2, ultimately leading to proteasome-mediated degradation.

### LZTS2 interacts with HAUSP

**Fig. 3:**
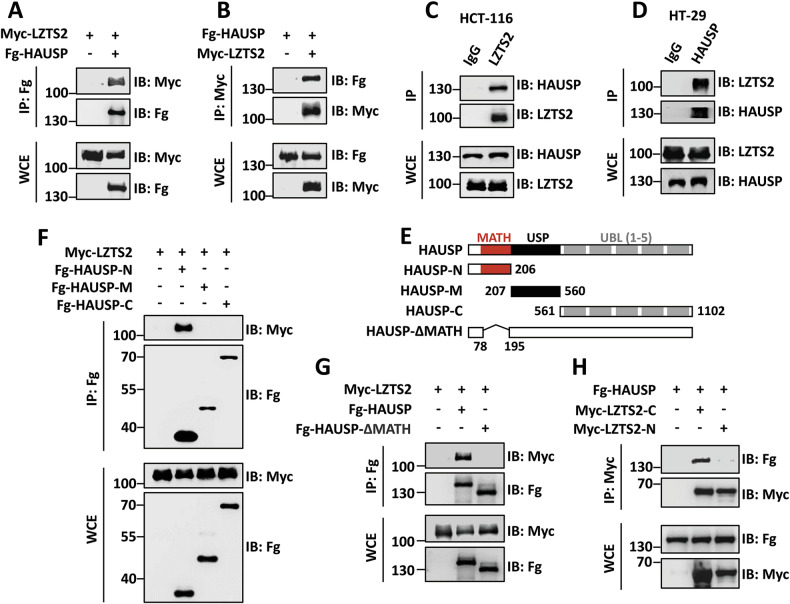
HAUSP binds to LZTS2 through its MATH domain. **A** Immunoblots of immunoprecipitates (IP, top two panels) or whole cell extracts (WCE, bottom two panels) from 293 T cells expressing the indicated plasmids. Of note, Fg-HAUSP was able to pull down Myc-LZTS2. **B** Myc-LZTS2 could immunoprecipitate Fg-HAUSP. **C** Endogenous LZTS2 interacted with endogenous HAUSP in HCT-116 cells. **D** Endogenous HAUSP immunoprecipitated endogenous LZTS2 in HT-29 cells. **E** Schematic drawings show the domains in HAUSP and the truncated mutants used in the following studies. **F** Fg-HAUSP-N could, but Fg-HAUSP-M and Fg-HAUSP-C could not pull down Myc-LZTS2. **G** Compared with Fg-HAUSP, Fg-HAUSP-ΔMATH failed to interact with Myc-LZTS2. (**H**) Fg-HAUSP interacted with Myc-LZTS2-C, not Myc-LZTS2-N.

Similar to other protein modifications, ubiquitination is also a reversible process due to the presence of DUBs [38]. A previous screen indicated HAUSP as the most probable LZTS2-interacting protein, ranking highest among all potential candidates. This suggests that HAUSP may have the ability to reverse the ubiquitination of LZTS2. To investigate this, we first examined the interaction between HAUSP and LZTS2. Co-IP assays revealed that Myc-LZTS2 reciprocally bound to Fg-HAUSP in 293 T cells (Fig. 3A-B). The interaction between HAUSP and LZTS2 was also observed at endogenous levels in both HCT-116 and HT-29 cells (Fig. 3C-D), confirming a reliable interaction between HAUSP and LZTS2.

HAUSP contains a MATH domain at the N-terminus, a ubiquitin specific protease (USP) domain in the middle, and five tandem ubiquitin-like (UBL) domains in its C-terminus [39]. Previous studies have revealed that HAUSP recognizes substrates through the MATH and UBL domains [40–42]. To identify which domain of HAUSP is involved in the interaction with LZTS2, we generated three truncated constructs and performed Co-IP experiments (Fig. 3E). The results showed that only the N-terminus of HAUSP could pull down LZTS2 (Fig. 3F), indicating the significance of the MATH domain in this interaction. Consistently, deleting the MATH domain of HAUSP abolished its binding to LZTS2 (Fig. 3G). Additionally, LZTS2 bound to HAUSP via its C-terminal (Fig. 3H).

### HAUSP deubiquitinates and stabilizes LZTS2

**Fig. 4:**
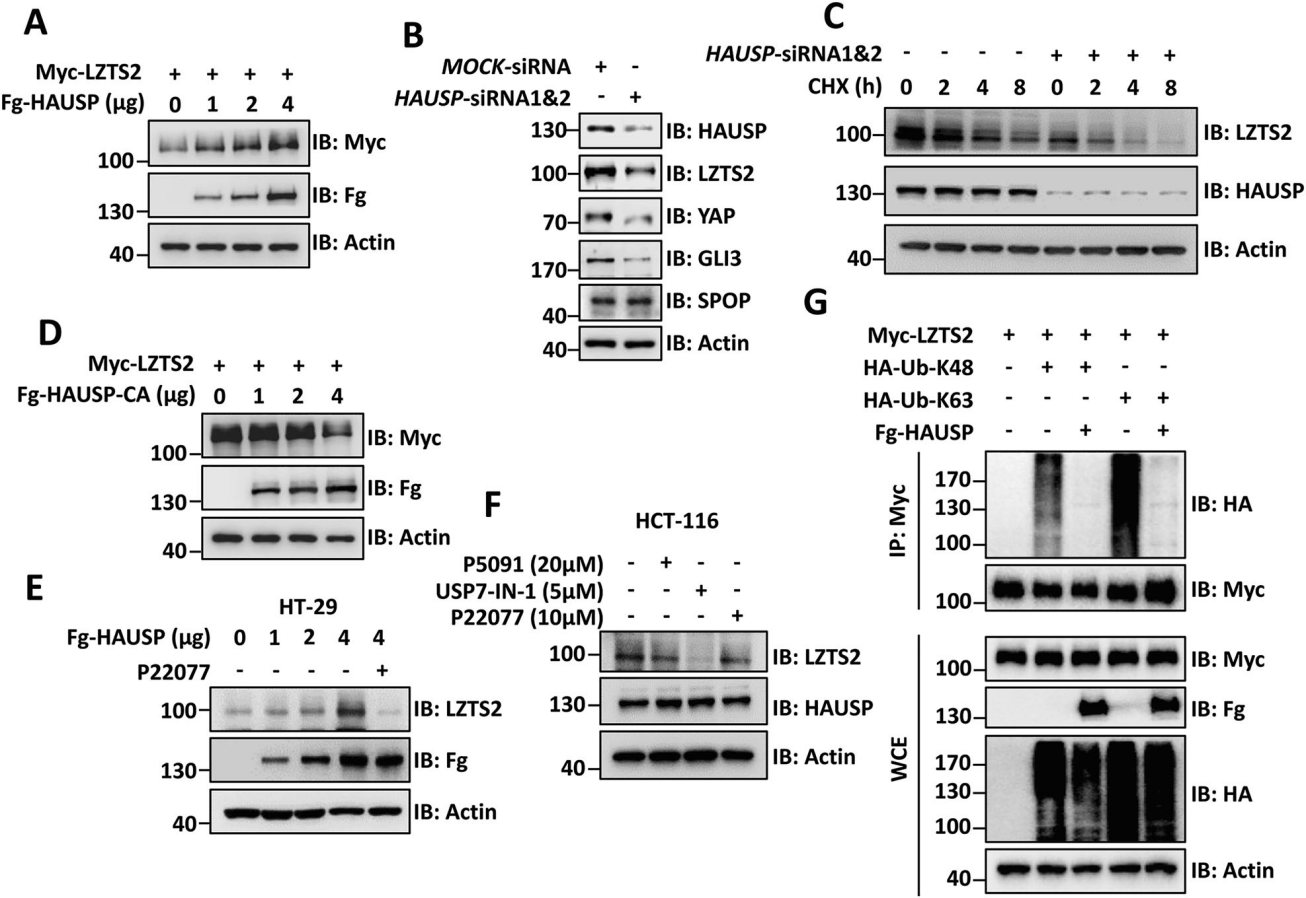
HAUSP removes ubiquitin chains from LZTS2 to stabilize it. **A** HAUSP increased Myc-LZTS2 protein in a dose-dependent manner in 293 T cells. **B** Knockdown of HAUSP decreased endogenous LZTS2, YAP and GLI3 protein levels in HCT-116 cells. **C** 293 T cells were treated with CHX (20 μg/ml) for the indicated times. Of note, knockdown of HAUSP promoted the degradation of endogenous LZTS2. **D** The deubiquitinase-deficiency mutant HAUSP-CA failed to increase Myc-LZTS2 protein. **E** HAUSP enhanced endogenous LZTS2 protein in a dose-dependent manner in HT-29 cells, which was abolished by treatment with 10 μM of HAUSP inhibitor P22077 for 24 h. **F** Treatment with three HAUSP inhibitors (P5091, USP7-IN-1, or P22077) at the indicated concentrations for 24 h apparently downregulated endogenous LZTS2 protein in HCT-116 cells. **G** Immunoblots of immunoprecipitates (top two panels) or whole cell extracts (bottom three panels) from 293 T cells transfected with the indicated plasmids. HAUSP suppressed K48- and K63-linked polyubiquitination on LZTS2. Above all, Actin acts as a loading control.

Given that HAUSP is a well-known deubiquitinating enzyme that plays a pivotal role in controlling protein stability, we suspected that LZTS2 might be a substrate of HAUSP. When Fg-HAUSP was overexpressed in 293 T cells, there was a noticeable increase in the levels of Myc-LZTS2 (Figs. 4A and S2D). Conversely, knocking down endogenous HAUSP with siRNA reduced the protein levels of LZTS2, as well as two well-characterized HAUSP substrates (YAP and GLI3) (Fig. 4B). Moreover, HAUSP knockdown led to a shorter half-life of LZTS2 protein compared to the control group (Fig. 4C).

As HAUSP typically interacts with the substrates to remove ubiquitin chains from them, we conducted experiments to investigate the importance of the deubiquitinase activity in stabilizing LZTS2. We created a mutant form of HAUSP (HAUSP-CA) with a deficiency in deubiquitinase activity by replacing the cysteine residue at 250 with alanine [43]. Our results showed that HAUSP-CA had a dominant negative effect, leading to a dose-dependent decrease in Myc-LZTS2 levels (Figs. 4D and S2E). Additionally, treatment with the HAUSP enzymatic activity inhibitor P22077 [44] effectively blocked the ability of Fg-HAUSP to increase endogenous LZTS2 abundance in HT-29 cells (Fig. 4E). Validation using two other HAUSP inhibitors, P5091 [45] and USP7-IN-1 [46], also resulted in decreased endogenous LZTS2 protein levels, without affecting HAUSP levels (Fig. 4F). These findings highlight the essential role of deubiquitinase activity in HAUSP-mediated stabilization of LZTS2 in CRC cells. Following this, we sought to determine if HAUSP deubiquitinates LZTS2 through cell-based ubiquitination experiments. Given the above findings that SPOP can enhance both K48- and K63-linked polyubiquitination of LZTS2, we explored whether HAUSP can remove these two types of polyubiquitin chains from LZTS2. Our findings indicated that HAUSP reduced both K48- and K63-linked ubiquitination on LZTS2 (Fig. 4G). In summary, HAUSP interacts with LZTS2 to exert its deubiquitination activity on LZTS2, leading to the stabilization of LZTS2.

### SPOP and HAUSP bidirectionally regulate LZTS2 ubiquitination

**Fig. 5:**
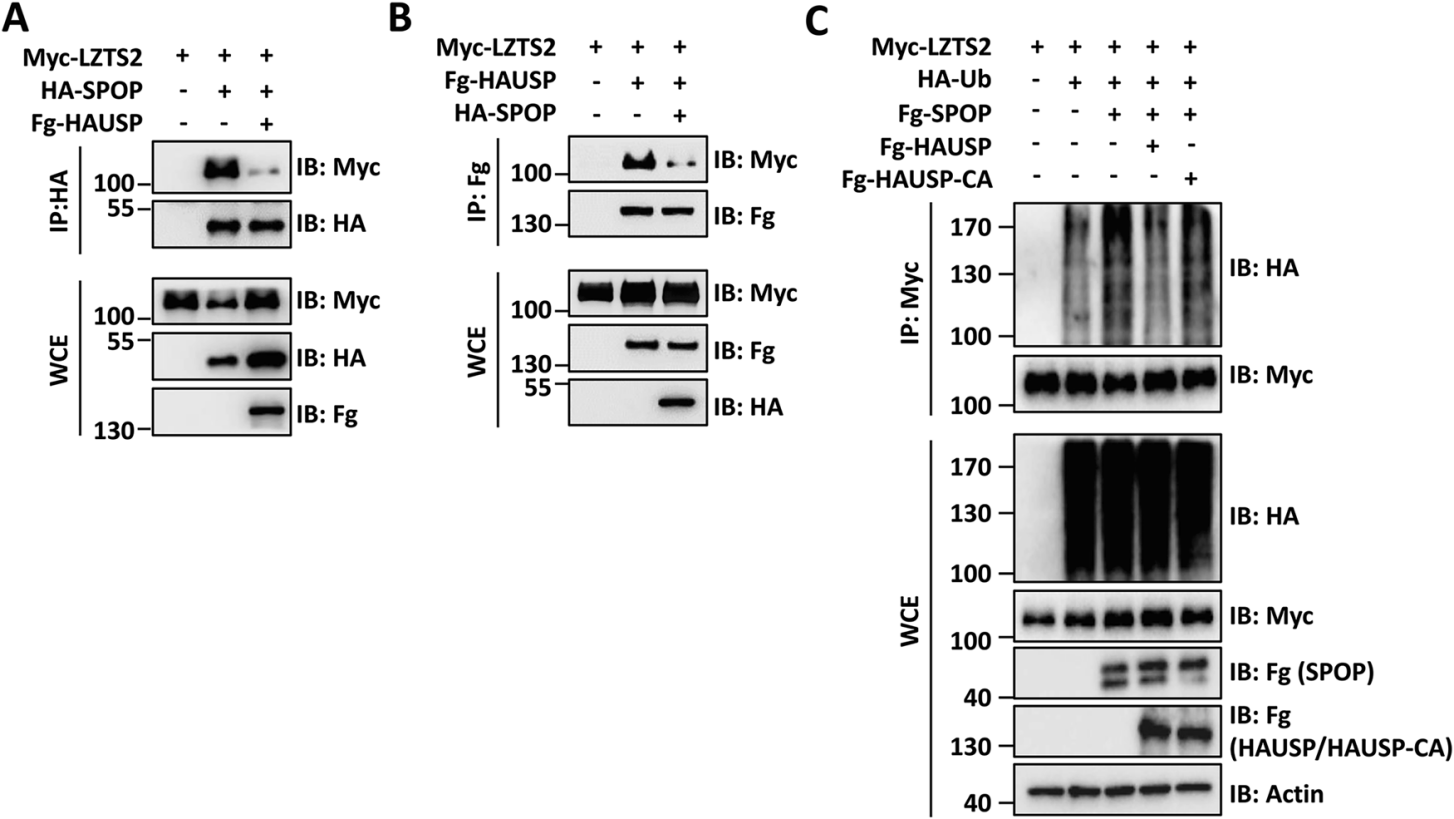
SPOP and HAUSP compete for binding LZTS2 to control its ubiquitination. **A** Fg-HAUSP weakened the interaction between HA-SPOP and Myc-LZTS2. **B** HA-SPOP enabled to decrease the affinity of Fg-HAUSP to Myc-LZTS2. **C** 293 T cells were transfected with the indicated plasmids and treated with 25 μM of MG132 for 6 h before cell harvesting. HAUSP, but not HAUSP-CA, antagonized SPOP-induced ubiquitination of LZTS2. Actin acts as a loading control.

The results mentioned above have shown that both SPOP and HAUSP interact with the C-terminus of LZTS2 (Figs. 1I and 3H), suggesting a potential competition between SPOP and HAUSP for binding to LZTS2. To test this possibility, we examined the strength of the interaction between SPOP and LZTS2 in the absence or presence of HAUSP, as well as vice versa. Our findings revealed that the presence of HAUSP weakened the binding of SPOP to LZTS2 (Fig. 5A), while overexpression of SPOP also decreased the interaction between HAUSP and LZTS2 (Fig. 5B), demonstrating that SPOP competes with HAUSP to interact with LZTS2. To confirm that the competition takes place at the binding level rather than affecting LZTS2 stability, we performed Co-IP assays using the N-terminal fragments of SPOP and HAUSP, both of which bind LZTS2 without altering its stability. The findings revealed that HAUSP-N and SPOP-N mutually interfere with each other’s interaction with LZTS2 (Fig. S3A), providing direct evidence of competitive binding. For a deeper examination of whether SPOP and HAUSP compete for the same binding sites on LZTS2, we utilized AlphaFold and PyMOL to predict the binding interfaces of both SPOP-N and HAUSP-N with LZTS2-C. Structural modeling revealed that the binding sites for SPOP-N and HAUSP-N on LZTS2-C are spatially adjacent and both include residue Q106 (Fig. S3B-C). Furthermore, the ubiquitination of LZTS2 triggered by SPOP was effectively reversed by co-transfecting HAUSP, rather than HAUSP-CA (Fig. 5C).

The delicate balance between ubiquitination and deubiquitination is often disturbed in various types of tumors [20]. Mutations in the MATH domain of SPOP are frequently observed in human cancer specimens, leading to changes in interactions between SPOP and its substrates [47, 48]. Studies have shown that cancer-associated mutations in SPOP can result in abnormal activation of oncogenic pathways, resulting in cancer progression [26–29]. We investigated whether cancer-derived mutations in SPOP affect its interaction with LZTS2. Co-IP results showed that the endometrial cancer (EC)-derived SPOP mutants (E47K, M117I and R121Q) exhibited similar interactions with LZTS2 as wild-type SPOP (Fig. S3D). However, the hepatocellular carcinoma (HCC)-related SPOP mutation, SPOP-M35L, showed a more robust affinity with LZTS2 than wild-type SPOP (Fig. S3E). Furthermore, we identified three CRC-related SPOP mutations (F32L, S54T and A61T) in The Cancer Genome Atlas (TCGA). Subsequent binding experiments confirmed that these mutants also exhibited a stronger affinity for LZTS2 (Fig. S3F). These findings suggest that mutations in the MATH domain of SPOP lead to distinct functional consequences in a context-dependent manner. Additionally, we identified two point-mutations, R93Q and M102L, in the MATH domain of HAUSP in CRC patient samples from TCGA. Surprisingly, these mutations weakened the binding of HAUSP to LZTS2 (Fig. S3G). Taken together, tumor-derived mutations in SPOP and HAUSP alter their interactions with the tumor suppressor LZTS2, potentially contributing to tumor development.

### SPOP and HAUSP regulate LZTS2’s inhibitory effect on Wnt pathway

**Fig. 6:**
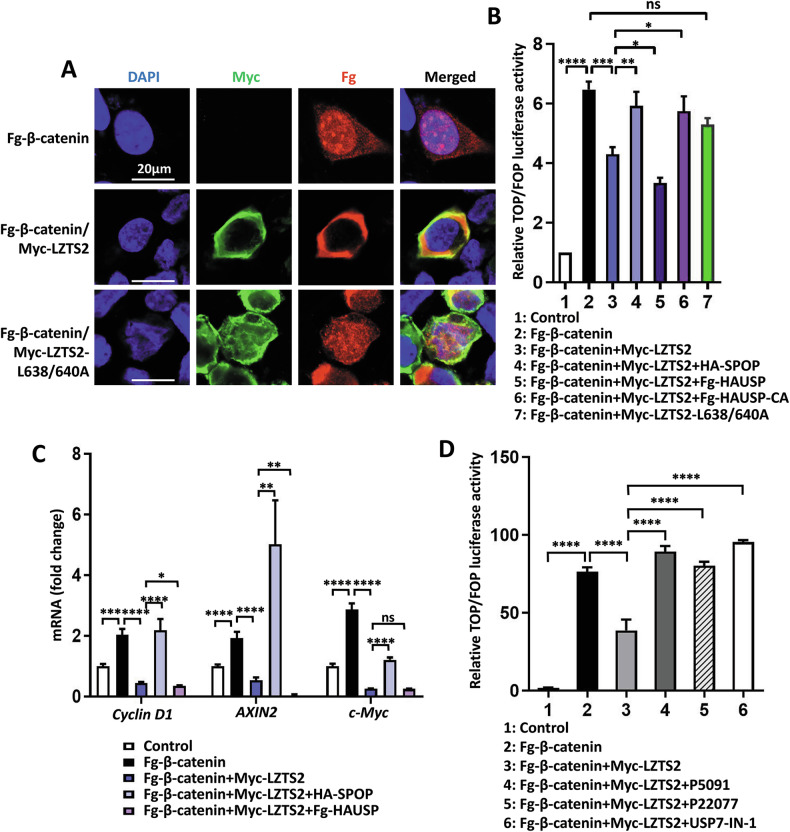
SPOP and HAUSP regulate the Wnt pathway activity through LZTS2. **A** 293 T cells expressing indicated constructs were stained to show Fg-β-catenin (red), Myc-LZTS2 or LZTS2-L638/640 A (green), and DAPI (blue). Of note, Fg-β-catenin was mainly localized in the nucleus, but this localization was blocked by LZTS2, not by LZTS2-L638/640 A. DAPI staining marks the cell nuclei. Scale bars: 20 μm for all images. **B** The relative TOP/FOP luciferase activity was detected by dual luciferase reporter assays in 293 T cells expressing the indicated constructs. **C** The relative mRNA levels of β-catenin target genes were detected by qRT-PCR. **D** The relative TOP/FOP luciferase activity was detected in 293 T cells expressing the indicated constructs with or without HAUSP inhibitors treatment.

The main function of LZTS2 is to bind β-catenin and prevent its nuclear accumulation, thereby negatively impacting the Wnt pathway [15]. Our above data showed that SPOP and HAUSP modulate LZTS2 stability, so it is necessary to explore their roles in the Wnt pathway. Immunofluorescence assays showed that co-expression of Myc-LZTS2 with Fg-β-catenin led to cytoplasmic accumulation of β-catenin (Fig. 6A). However, co-expression of a nuclear export signal deficient form of LZTS2 (LZTS2-L638/640 A) attenuated the cytoplasmic localization of LZTS2 to β-catenin (Fig. 6A), highlighting the role of LZTS2 in regulating the subcellular localization of β-catenin.

Two sets of experiments were conducted to investigate the involvement of SPOP and HAUSP in modulating the Wnt pathway. In the first experiment, the TOP/FOP luciferase assay was carried out using β-catenin to drive luciferase expression from a DNA fragment containing TCF/LEF-responsive element. LZTS2 reduced β-catenin-driven TOP/FOP activity, which was rescued by SPOP co-expression (Fig. 6B). Conversely, co-transfection with HAUSP, but not HAUSP-CA, exacerbated the effect of LZTS2 in decreasing TOP/FOP activity (Fig. 6B). However, when LZTS2-L638/640 A was co-expressed with β-catenin, it did not decrease TOP/FOP activity. In the second experiment, RT-qPCR was conducted to examine the expression of β-catenin target genes *Cyclin D1*, *AXIN2* and *c-Myc* in HCT116 cells. As expected, β-catenin was able to activate the expression of these target genes, which was suppressed by co-expressing LZTS2 (Fig. 6C). The inhibition of LZTS2 on β-catenin was relieved by SPOP, but strengthened by HAUSP (Fig. 6C). On the other hand, treatment with HAUSP inhibitors effectively removed LZTS2’s inhibition on β-catenin-driven TOP/FOP activity (Fig. 6D). In conclusion, SPOP and HAUSP regulate Wnt/β-catenin pathway activity through LZTS2.

### SPOP and HAUSP control the anti-tumor capacity of LZTS2

**Fig. 7:**
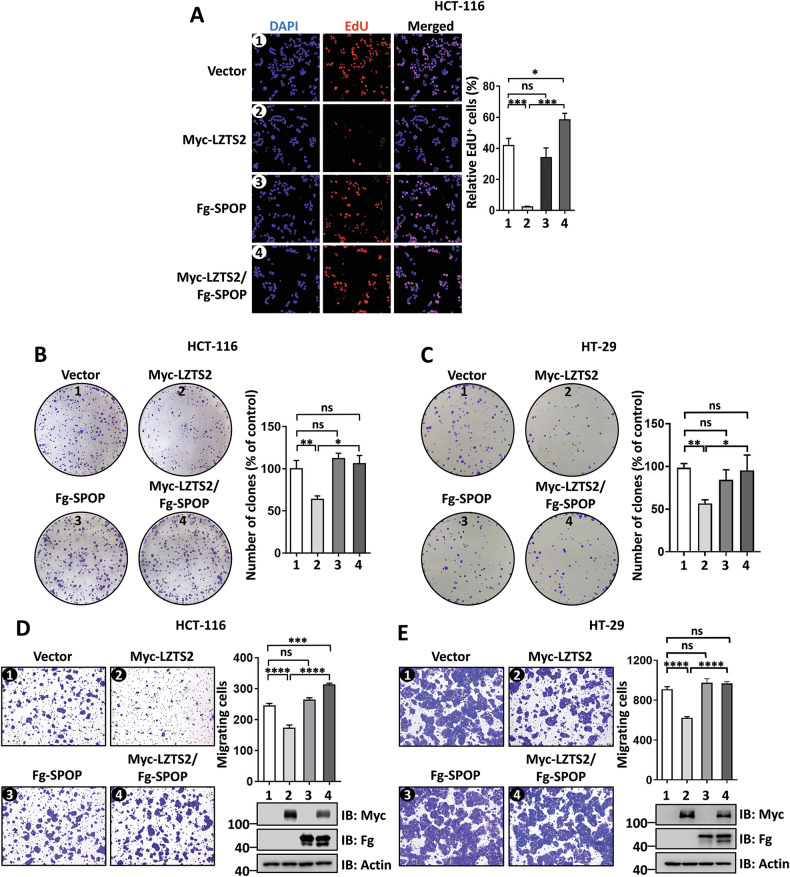
SPOP blocks the inhibition of LZTS2 on CRC cell migration and proliferation. **A** EdU incorporation assay showed that SPOP co-expression could restore LZTS2-suppressed HCT-116 cell proliferation. Quantitative analysis was shown on the right. The colony formation assays showed the proliferation of HCT-116 (**B**) and HT-29 cells (**C**) expressing indicated constructs. Quantitative analyses were shown on the right. The migrative abilities of HCT-116 (**D**) and HT-29 cells (**E**) transfected with the specified constructs were assessed by transwell assays. The expressions of constructs were examined by immunoblotting. Quantitative analyses were shown on the right.

To evaluate the functional relevance of SPOP-mediated LZTS2 ubiquitination, we selected colorectal cancer (CRC) since CRC cells are known to be sensitive to the Wnt pathway [49, 50]. Previous studies have demonstrated that LZTS2 plays a role in inhibiting tumor growth in CRC by sequestering β-catenin in the cytoplasm [15, 51, 52]. Consistent with this, overexpression of LZTS2 reduced EdU incorporation in HCT-116 cells (Fig. 7A), while knockdown of LZTS2 increased EdU incorporation (Fig. S4A-B), indicating that LZTS2 suppresses HCT-116 cell proliferation. Moreover, the decrease in EdU incorporation caused by LZTS2 was reversed by co-expressing SPOP (Fig. 7A). Nevertheless, overexpression of SPOP alone did not influence cell proliferation, as it functions as an E3 ubiquitin ligase adaptor whose function depends on the presence and abundance of substrates. In line with this, high SPOP expression was found to be linked to poorer overall survival in CRC patients, particularly in the LZTS2-high (LZTS2^H^) subgroup, but not in the LZTS2-low (LZTS2^L^) subgroup (Fig. S4C). In addition, the colony formation assay was carried out to assess cell growth capacity. LZTS2 suppressed colony formation of HCT-116 cells, but this inhibition was rescued when SPOP was co-expressed (Fig. 7B). This finding was confirmed in another CRC cell line, HT-29 (Fig. 7C). The transwell assay was utilized to evaluate cell migration, another key feature of tumor cells. Overexpression of LZTS2 suppressed cell migration in both HCT-116 and HT-29 cells, which was completely reversed by co-expressing SPOP (Fig. 7D-E). Taken together, these results demonstrate that SPOP counteracts the suppression of CRC cell migration and proliferation caused by LZTS2.

**Fig. 8:**
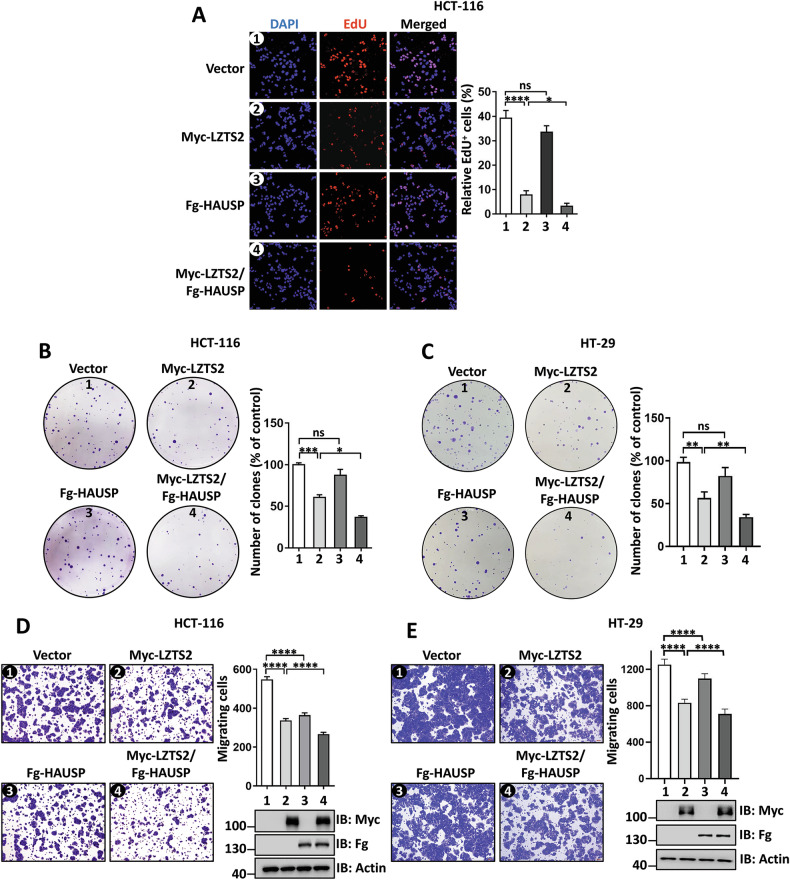
HAUSP enhances the LZTS2-inhibited CRC cell migration and proliferation. **A** The EdU incorporation assay revealed that HAUSP co-expression could boost LZTS2-suppressed HCT-116 cell proliferation. Quantitative analysis was shown on the right. The colony formation assays revealed the proliferation of HCT-116 (**B**) and HT-29 cells (**C**) with indicated transfection. Quantitative analyses were shown on the right. The migration of HCT-116 (**D**) and HT-29 cells (**E**) transfected with the specified constructs was tested by transwell assays. The expressions of constructs were examined by immunoblotting. Quantitative analyses were shown on the right.

We next sought to examine the functional significance of HAUSP-mediated LZTS2 stabilization in CRC cell proliferation and migration. The EdU incorporation assay revealed that LZTS2-induced cell arrest was aggravated by co-expressing HAUSP in HCT-116 cells (Fig. 8A). Consistently, the colony formation assay showed that HAUSP augmented LZTS2-caused decrease in colony numbers in HCT-116 and HT-29 cells (Fig. 8B-C). In addition, the inhibition of cell migration by LZTS2 was further strengthened through co-expressing HAUSP in CRC cells (Fig. 8D-E), while this inhibition was rescued by treatment with HAUSP inhibitors (Fig. S4D). Taken together, these findings suggest that SPOP and HAUSP regulate the anti-tumor capacity of LZTS2 in CRC cell lines possibly through modulating its stability.

## Discussion

**Fig. 9:**
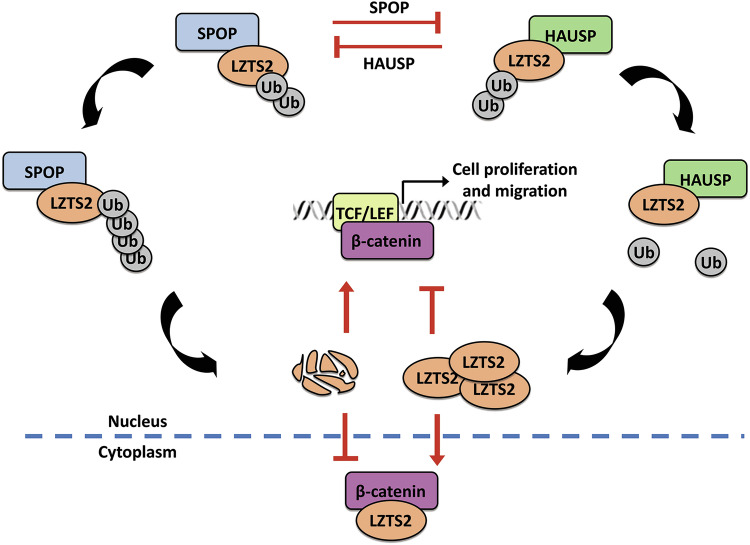
A proposed model for SPOP and HAUSP regulating LZTS2. SPOP and HAUSP compete to bind to LZTS2, serving as opposing ubiquitin-modifying enzymes that regulate the degradation of LZTS2. SPOP counters while HAUSP supports the cytoplasmic retention of β-catenin mediated by LZTS2, thereby regulating CRC cell migration and proliferation.

CRC is a malignant tumor with an extremely high incidence and mortality rate worldwide [1]. Its pathogenesis is still unclear, which restricts clinical diagnosis and treatment. Overactivation of the Wnt pathway is a key mechanism for CRC initiation and progression [53]. Recently, several studies have uncovered that LZTS2 suppresses the Wnt pathway through inhibiting the nuclear localization of β-catenin, an important transducer of this pathway [15]. Thus, overexpression of LZTS2 is able to suppress Wnt-driven CRC progression [52]. LZTS2 is phosphorylated by the kinase PLK1 to weaken its interaction with β-catenin, culminating in activating the Wnt pathway [16]. However, whether LZTS2 is underwent regulation by other post-translation modifications is still unknown. Here, we reveal that LZTS2 is reversibly ubiquitinated by the E3 ligase SPOP and the deubiquitinase HAUSP, together modulating the stability of LZTS2. Moreover, SPOP and HAUSP compete for LZTS2 binding, leading to opposing effects on the ubiquitination and protein levels of LZTS2 (Fig. 9). This balance ultimately determines LZTS2’s ability to sequester β-catenin in the cytoplasm, thereby influencing Wnt pathway activity. As a result, SPOP counteracts while HAUSP boosts the tumor-suppressive properties of LZTS2 in CRC cell proliferation and migration (Fig. 9).

*LZTS*2 and another tumor suppressor *PTEN* are both located on chromosome 10, with a distance of ~15 Mb, frequently deleted concurrently in clinical tumor samples [54]. Compared with mice lacking either *PTEN* or *LZTS*2 alone, mice with both *LZTS*2 and *PTEN* knockout show earlier onset of prostate cancer and faster tumor progression, suggesting that LZTS2 and PTEN exert a synergistic tumor suppressor effect [17]. LZTS2 and PTEN may have functional relevance in suppressing tumorigenesis. Previous research has uncovered that SPOP contributes to tumorigenesis by ubiquitinating and destabilizing PTEN in clear cell renal cell carcinoma [55]. Our study demonstrates that SPOP promotes CRC progression through degrading LZTS2. These indicate that SPOP plays a dual role in tumorigenesis, targeting both LZTS2 and PTEN, which may collectively enhance the progression of tumors. It will be interesting to explore how SPOP chooses between LZTS2 and PTEN for degradation in tumorigenesis, and if LZTS2 and PTEN compete for binding to the E3 ligase SPOP.

Maintaining a balance between ubiquitination and deubiquitination modifications in the ubiquitin-proteasome system is critical for protein posttranslational regulation. Disruption of this balance has been connected to a variety of human cancers [56]. SPOP shows a high frequency of somatic mutations in several types of cancer [47, 48]. The majority of reported SPOP mutations are loss-of-function, leading to decreased affinity for its oncogenic substrates [26–29]. In our earlier research, we identify a gain-of-function mutation in liver cancer, known as M35L. This mutation reprograms SPOP from a tumor suppressor to an oncoprotein by heightening its interaction with the anti-tumor substrate, IRF2BP2 [29]. SPOP-M35L and CRC-derived SPOP mutants (F32L, S54T and A61T) display enhanced binding affinity to LZTS2, while EC-derived SPOP mutants (E47K, M117I and R121Q) have interactions with LZTS2 comparable to wild-type SPOP, indicating that distinct mutations result in different outcomes. Besides, we have identified two HAUSP mutants (R93Q, M102L) derived from CRC that show decreased affinity towards LZTS2. In the TCGA mutation profiles, we amazingly find that mutations on SPOP and HAUSP cluster in the MATH domain, which is responsible for binding substrates. Therefore, alterations in substrate affinity due to mutations in the substrate binding domain of SPOP and HAUSP are important mechanisms leading to tumorigenesis.

The abnormal activation of the Wnt/β-catenin pathway plays a key role in the initiation and progression of CRC [5]. In clinical CRC patient samples, mutations in components of this pathway, such as APC and β-catenin, result in enhanced transcription of target genes that support cell proliferation and survival [57]. Therefore, inhibiting the entry of β-catenin into the nucleus or reducing its level can be potential strategies for treating tumors caused by excessive Wnt pathway activation. Direct pharmacological targeting of β-catenin faces challenges due to its crucial roles in various physiological processes and the absence of clearly defined drug-binding pockets [58]. Our study proposes an alternative strategy to suppress β-catenin activity by increasing LZTS2 levels. By targeting the SPOP-HAUSP axis to enhance LZTS2 stabilization, we could potentially achieve a more feasible approach. Inhibiting SPOP pharmacologically or activating HAUSP may help restore LZTS2 levels and its tumor-suppressive role. Moreover, determining the mutational status of SPOP (F32L, S54T and A61T) or HAUSP (R93Q, M102L) may aid in identifying patients who are most likely to benefit from these strategies. Therefore, future studies should concentrate on developing specific disruptors of the SPOP-LZTS2 interaction and stabilizers of the HAUSP-LZTS2 complex.

## Materials and methods

### Cell lines, transfection and immunoblotting

HEK-293T (293 T), HCT-116 and HT-29 cell lines were purchased from the American Type Culture Collection (ATCC). 293 T cells were cultured in Dulbecco’s modified Eagle’s medium (DMEM) supplemented with 10% fetal bovine serum (FBS). HCT-116 and HT-29 cells were cultured in RPMI-1640 medium containing 10% FBS at 37°C and 5% CO_2_. All these cell lines underwent routine testing to rule out any mycoplasma contamination.

293 T cells were transfected using PEI (Sigma), while HCT-116 and HT-29 cells were transfected via Lipofectamine 3000 (Thermo Fisher Scientific) following the manufacturer’s instructions for 48 h. After transfection, the cells were harvested and lysed for immunoprecipitation (IP) and immunoblotting (IB) assays according to previous study described [59]. The antibodies used for IB or IP were as follows: rabbit anti-SPOP (ProteinTech), rabbit anti-LZTS2 (Proteintech), rabbit anti-YAP (ABclonal), rabbit anti-GLI3 (ABclonal), mouse anti-USP7 (ABclonal), mouse anti-Actin (Genscript), mouse anti-Myc (Santa Cruz), mouse anti-Fg (Sigma) and mouse anti-HA (Santa Cruz). In some experiments, MG132 (25 μM; Calbiochem) was added to cultured cells to inhibit proteasome activity, whereas NH_4_Cl (10 mM; Ambeed) was used to block lysosome function. For HAUSP inhibitors treatment, cells were treated by P22077 (#HY-13865, MedChemExpress), USP7-IN (#HY-16709, MedChemExpress) or P5091 (#SML0070, Sigma) at indicated concentrations for 24 h.

### DNA constructs

To generate constructs for transfection of LZTS2, SPOP, HAUSP, β-catenin, CUL3, RBX1 and Ub, the full-length coding sequences were amplified using CRC cells cDNA as the template, and cloned into the pcDNA3.1-Fg, pcDNA3.1-Myc, pcDNA3.1-HA or pGEX-4T-3 backbone vectors. Truncated fragments were amplified via PCR, and inserted into the corresponding backbone vectors. Point mutation constructs utilized in this study were generated through PCR-based site-directed mutagenesis in the context of Fg-SPOP, Fg-HAUSP, Myc-LZTS2 or HA-Ub, as described in our previous study [42]. Small interfering RNA (siRNA) for *MOCK*, *LZTS2* and *HAUSP* were purchased from Genepharma (Shanghai, China). The siRNA sequences used were as follows: *MOCK*-siRNA, 5’-UUC UCC GAA CGU GUC ACG UUU dTdT-3’, *LZTS2*-siRNA1, 5’-GGA GGA GAU CAC UGC UAC U dTdT-3’, *LZTS2*-siRNA2, 5’-GCA GCA GCU GAA AGA GUC U dTdT-3′, *HAUSP*-siRNA1, 5’-ACC CUU GGA CAA UAU UCC U dTdT-3’, *HAUSP*-siRNA2, 5′-AGU CGU UCA GUC GUC GUA U dTdT-3’.

### Immunofluorescence and confocal

Cell-based immunofluorescence assays were carried out as previously described [29]. For EdU incorporation assays, HCT-116 cells were transfected with indicated constructs and then seeded onto chamber slides. These cells were next incubated with 10 μM EdU (CellorLab) for 2 h before cell harvesting. The subsequent immunofluorescence steps were performed following the same protocol as described above.

### Luciferase reporter assay

The TOP and FOP TCF/LEF reporters were gifted from Prof. Chuyong Lin [60]. 293 T cells were transfected with the indicated plasmids, luciferase reporters, and Pol II-Renilla for 48 h. Luciferase activity was then measured using the Dual-Luciferase Reporter assay (Vazyme). Data were presented as means ± SEM of values from at least three experiments.

### RNA extraction and qRT-PCR

Total RNAs were isolated from HCT-116 cells after transfection with TRIzol (Invitrogen) following standard protocols. The RNA was reverse transcribed using HiScript® Q RT SuperMix with gDNA wiper (Vazyme) following the manufacturer’s instructions. The primer pairs used were as follows: *Cyclin D1*, 5’-CAT TGA ACA CTT CCT CTC CA-3’ (forward) and 5’-AAC TTC ACA TCT GTG GCA C-3’ (reverse); *AXIN2*, 5’-TTA TGC TTT GCA CTA CGT CCC TCC A-3’ (forward) and 5’-CGC AAC ATG GTC AAC CCT CAG AC-3′ (reverse); *c-Myc*, 5’-AAA CCT CCT CAC AGC CCA CT-3’ (forward) and 5’-GTT CCG TAG CTG TTC AAG TTT G-3’ (reverse); *ACTIN*, 5’-TGA CAT TAA GGA GAA GCT GTG CTA C-3′ (forward) and 5’-GAG TTG AAG GTA GTT TCG TGG ATG-3’ (reverse). Data are presented as means ± SEM of values from at least three repeats. Real-time PCR was performed using ChamQ SYBR® Color qPCR Master Mix (Vazyme) on ZY/VQ-100A (Yuanzan). Relative expressions of indicated genes were detected using the 2^-∆∆Ct^ method.

### In vitro ubiquitination assay

Briefly, GST-LZTS2 was expressed in and purified from *E. coli* BL21 by GSH magnetic agarose beads (Beyotime). 293 T cells were co-transfected with HA-SPOP, Fg-CUL3 and Myc-RBX1 for 48 h. These cells were next harvested and lysed by sonication in IP buffer (20 mM Tris-HCl pH 7.4, 150 mM NaCl, 5% glycerol, 1 mM TCEP and 1 × protease inhibitor cocktail). The SPOP-CUL3-RBX1 complex was purified by anti-HA M2 affinity gel and washed with IP buffer. For the in vitro ubiquitination assay, purified GST-LZTS2 was incubated with the SPOP-CUL3-RBX1 complex, E1 (Ubbiotech), E2 (Ubbiotech) and ubiquitin (Ubbiotech) in reaction buffer (Ubbiotech) supplemented with ATP at 30 °C for 2 h. The reaction products were then analyzed by western blotting.

### Transwell assay

At 48 h after transfection, a total of 2 × 10^5^ cells were seeded in the upper transwell chambers (BD Biosciences) containing 300 μL of serum-free medium. The lower chambers of a 24-well plate (Corning) were filled with 500 μL of medium containing 10% FBS. The transwell chambers were then incubated at 37 °C with 5% CO_2_ for 24 h. After incubation, the cells that migrated to the bottom surface were fixed with 20% methanol for 20 min. The cells were stained with 0.1% crystal violet (Sangon Biotech), and the cells on the upper surface of the inserts were carefully removed using cotton swabs. Finally, the stained cells were photographed and quantified under a microscope in 8 randomly chosen fields.

### Colony formation assay

To conduct the colony formation assay, single-cell suspensions were seeded into six-well plate at a density of 2000 cells per well and incubated at 37°C with 5% CO_2_ for 2 weeks. After incubation, the plates were washed with PBS, fixed with 4% formaldehyde, and stained with 0.1% crystal violet (Solarbio) for 1 h. Colonies containing more than 50 cells were then counted.

### AlphaFold structural modeling

Structural models of the SPOP-N/LZTS2-C and HAUSP-N/LZTS2-C complexes were predicted using AlphaFold3. Five models were generated for each complex. The representative model for each complex was selected based on optimal combined scores for the predicted local distance difference test (pLDDT) and predicted alignment error (PAE). All structural visualizations were performed using PyMOL.

### Statistical analysis

Statistical analyses were performed using GraphPad Prism software (GraphPad Software Inc., La Jolla, CA). All data presented in this study were representative of three or more independent replicates and were displayed as means ± SEM. Statistical significance was determined through a two-tailed unpaired Student’s *t*-test using Prism software (GraphPad), with significance values set at *P < 0.05, **P < 0.01, ***P < 0.001, ****P < 0.0001 and ns (not significant, *P* > 0.05).

## Supplementary information

Supplementary Information

## Data Availability

All relevant data are available from the corresponding author upon reasonable request.

## References

[1] Patel SG, Dominitz JA. Screening for colorectal cancer. Ann Intern Med. 2024;177:Itc49–itc64. 38588547 10.7326/AITC202404160

[2] Yan H, Talty R, Aladelokun O, Bosenberg M, Johnson CH. Ferroptosis in colorectal cancer: a future target?. Br J Cancer. 2023;128:1439–51. 36703079 10.1038/s41416-023-02149-6 PMC10070248

[3] Xie YH, Chen YX, Fang JY. Comprehensive review of targeted therapy for colorectal cancer. Signal Transduct Target Ther. 2020;5:22. 32296018 10.1038/s41392-020-0116-z PMC7082344

[4] Ohishi T, Kaneko MK, Yoshida Y, Takashima A, Kato Y, Kawada M. Current targeted therapy for metastatic colorectal cancer. Int J Mol Sci. 2023;24. 10.3390/ijms24021702 PMC9864602 36675216

[5] Zhao H, Ming T, Tang S, Ren S, Yang H, Liu M, et al. Wnt signaling in colorectal cancer: pathogenic role and therapeutic target. Mol Cancer. 2022;21:144. 35836256 10.1186/s12943-022-01616-7 PMC9281132

[6] Bodine PV. Wnt signaling control of bone cell apoptosis. Cell Res. 2008;18:248–53. 18212734 10.1038/cr.2008.13

[7] Stamos JL, Weis WI. The β-catenin destruction complex. Cold Spring Harb Perspect Biol. 2013;5:a007898. 23169527 10.1101/cshperspect.a007898 PMC3579403

[8] Clevers H. Wnt/beta-catenin signaling in development and disease. Cell. 2006;127:469–80. 17081971 10.1016/j.cell.2006.10.018

[9] Daniels DL, Weis WI. Beta-catenin directly displaces Groucho/TLE repressors from Tcf/Lef in Wnt-mediated transcription activation. Nat Struct Mol Biol. 2005;12:364–71. 15768032 10.1038/nsmb912

[10] Liu J, Xiao Q, Xiao J, Niu C, Li Y, Zhang X, et al. Wnt/β-catenin signalling: function, biological mechanisms, and therapeutic opportunities. Signal Transduct Target Ther. 2022;7:3. 34980884 10.1038/s41392-021-00762-6 PMC8724284

[11] Heng WS, Cheah SC Identification of phytochemical-based β-catenin nuclear localization inhibitor in NSCLC: differential targeting population from member of isothiocyanates. Molecules. 2021;26. 10.3390/molecules26020399 PMC7828655 33451160

[12] Wang W, Wen Q, Luo J, Chu S, Chen L, Xu L, et al. Suppression of β-catenin nuclear translocation by CGP57380 decelerates poor progression and potentiates radiation-induced apoptosis in nasopharyngeal carcinoma. Theranostics. 2017;7:2134–249. 28656063 10.7150/thno.17665 PMC5485425

[13] Griffin JN, Del Viso F, Duncan AR, Robson A, Hwang W, Kulkarni S, et al. RAPGEF5 regulates nuclear translocation of β-catenin. Dev Cell. 2018;44:248–260.e244. 29290587 10.1016/j.devcel.2017.12.001 PMC5818985

[14] Cabeza-Arvelaiz Y, Thompson TC, Sepulveda JL, Chinault AC. LAPSER1: a novel candidate tumor suppressor gene from 10q24.3. Oncogene. 2001;20:6707–17. 11709705 10.1038/sj.onc.1204866

[15] Thyssen G, Li TH, Lehmann L, Zhuo M, Sharma M, Sun Z. LZTS2 is a novel beta-catenin-interacting protein and regulates the nuclear export of beta-catenin. Mol Cell Biol. 2006;26:8857–67. 17000760 10.1128/MCB.01031-06 PMC1636836

[16] Liu R, Zhou D, Yu B, Zhou Z. Phosphorylation of LZTS2 by PLK1 activates the Wnt pathway. Cell Signal. 2024;120:111226. 38740232 10.1016/j.cellsig.2024.111226

[17] Yu EJ, Hooker E, Johnson DT, Kwak MK, Zou K, Luong R, et al. LZTS2 and PTEN collaboratively regulate ß-catenin in prostatic tumorigenesis. PLoS One. 2017;12:e0174357. 28323888 10.1371/journal.pone.0174357 PMC5360334

[18] Xu S, Li Y, Lu Y, Huang J, Ren J, Zhang S, et al. LZTS2 inhibits PI3K/AKT activation and radioresistance in nasopharyngeal carcinoma by interacting with p85. Cancer Lett. 2018;420:38–48. 29409973 10.1016/j.canlet.2018.01.067

[19] Peng Y, Greenland NY, Lang UE, Stohr BA. LZTS2: A novel and independent prognostic biomarker for clear cell renal cell carcinoma. Pathol Res Pr. 2022;232:153831. 10.1016/j.prp.2022.153831 35287088

[20] Dewson G, Eichhorn PJA, Komander D. Deubiquitinases in cancer. Nat Rev Cancer. 2023;23:842–62. 37935888 10.1038/s41568-023-00633-y

[21] Cruz Walma DA, Chen Z, Bullock AN, Yamada KM. Ubiquitin ligases: guardians of mammalian development. Nat Rev Mol Cell Biol. 2022;23:350–67. 35079164 10.1038/s41580-021-00448-5

[22] Zhuang M, Calabrese MF, Liu J, Waddell MB, Nourse A, Hammel M, et al. Structures of SPOP-substrate complexes: insights into molecular architectures of BTB-Cul3 ubiquitin ligases. Mol Cell. 2009;36:39–50. 19818708 10.1016/j.molcel.2009.09.022 PMC2847577

[23] Chen MH, Wilson CW, Li YJ, Law KK, Lu CS, Gacayan R, et al. Cilium-independent regulation of Gli protein function by Sufu in Hedgehog signaling is evolutionarily conserved. Genes Dev. 2009;23:1910–28. 19684112 10.1101/gad.1794109 PMC2725943

[24] Geng C, Kaochar S, Li M, Rajapakshe K, Fiskus W, Dong J, et al. SPOP regulates prostate epithelial cell proliferation and promotes ubiquitination and turnover of c-MYC oncoprotein. Oncogene. 2017;36:4767–77. 28414305 10.1038/onc.2017.80 PMC5887163

[25] Groner AC, Cato L, de Tribolet-Hardy J, Bernasocchi T, Janouskova H, Melchers D, et al. TRIM24 is an oncogenic transcriptional activator in prostate cancer. Cancer Cell. 2016;29:846–58. 27238081 10.1016/j.ccell.2016.04.012 PMC5124371

[26] Ju LG, Zhu Y, Long QY, Li XJ, Lin X, Tang SB, et al. SPOP suppresses prostate cancer through regulation of CYCLIN E1 stability. Cell Death Differ. 2019;26:1156–68. 30237511 10.1038/s41418-018-0198-0 PMC6748103

[27] Wang X, Jin J, Wan F, Zhao L, Chu H, Chen C, et al. AMPK promotes SPOP-mediated NANOG degradation to regulate prostate cancer cell stemness. Dev Cell. 2019;48:345–360.e347. 30595535 10.1016/j.devcel.2018.11.033 PMC7523188

[28] Zhang J, Bu X, Wang H, Zhu Y, Geng Y, Nihira NT, et al. Cyclin D-CDK4 kinase destabilizes PD-L1 via cullin 3-SPOP to control cancer immune surveillance. Nature. 2018;553:91–5. 29160310 10.1038/nature25015 PMC5754234

[29] Deng Y, Ding W, Ma K, Zhan M, Sun L, Zhou Z, et al. SPOP point mutations regulate substrate preference and affect its function. Cell Death Dis. 2024;15:172. 38409107 10.1038/s41419-024-06565-1 PMC10897488

[30] Hjorth-Jensen K, Maya-Mendoza A, Dalgaard N, Sigurðsson JO, Bartek J, Iglesias-Gato D, et al. SPOP promotes transcriptional expression of DNA repair and replication factors to prevent replication stress and genomic instability. Nucleic Acids Res. 2018;46:9891. 30165642 10.1093/nar/gky788 PMC6182152

[31] Marzahn MR, Marada S, Lee J, Nourse A, Kenrick S, Zhao H, et al. Higher-order oligomerization promotes localization of SPOP to liquid nuclear speckles. EMBO J. 2016;35:1254–75. 27220849 10.15252/embj.201593169 PMC4910529

[32] Zhang H, Jin X, Huang H. Deregulation of SPOP in cancer. Cancer Res. 2023;83:489–99. 36512624 10.1158/0008-5472.CAN-22-2801

[33] Cheng J, Guo J, Wang Z, North BJ, Tao K, Dai X, et al. Functional analysis of Cullin 3 E3 ligases in tumorigenesis. Biochim Biophys Acta Rev Cancer. 2018;1869:11–28. 29128526 10.1016/j.bbcan.2017.11.001 PMC7076836

[34] Dubiel W, Dubiel D, Wolf DA, Naumann M. Cullin 3-based ubiquitin ligases as master regulators of mammalian cell differentiation. Trends Biochem Sci. 2018;43:95–107. 29249570 10.1016/j.tibs.2017.11.010 PMC5801050

[35] Furukawa M, He YJ, Borchers C, Xiong Y. Targeting of protein ubiquitination by BTB-Cullin 3-Roc1 ubiquitin ligases. Nat Cell Biol. 2003;5:1001–7. 14528312 10.1038/ncb1056

[36] Hollstein PE, Cichowski K. Identifying the Ubiquitin Ligase complex that regulates the NF1 tumor suppressor and Ras. Cancer Discov. 2013;3:880–93. 23661552 10.1158/2159-8290.CD-13-0146 PMC3881282

[37] Ohtake F, Saeki Y, Ishido S, Kanno J, Tanaka K. The K48-K63 branched ubiquitin chain regulates NF-κB signaling. Mol Cell. 2016;64:251–66. 27746020 10.1016/j.molcel.2016.09.014

[38] Lange SM, Armstrong LA, Kulathu Y. Deubiquitinases: from mechanisms to their inhibition by small molecules. Mol Cell. 2022;82:15–29. 34813758 10.1016/j.molcel.2021.10.027

[39] Kim RQ, van Dijk WJ, Sixma TK. Structure of USP7 catalytic domain and three Ubl-domains reveals a connector α-helix with regulatory role. J Struct Biol. 2016;195:11–18. 27183903 10.1016/j.jsb.2016.05.005

[40] Saridakis V, Sheng Y, Sarkari F, Holowaty MN, Shire K, Nguyen T, et al. Structure of the p53 binding domain of HAUSP/USP7 bound to Epstein-Barr nuclear antigen 1 implications for EBV-mediated immortalization. Mol Cell. 2005;18:25–36. 15808506 10.1016/j.molcel.2005.02.029

[41] Cheng J, Yang H, Fang J, Ma L, Gong R, Wang P, et al. Molecular mechanism for USP7-mediated DNMT1 stabilization by acetylation. Nat Commun. 2015;6:7023. 25960197 10.1038/ncomms8023 PMC4432644

[42] Sun X, Ding Y, Zhan M, Li Y, Gao D, Wang G, et al. Usp7 regulates Hippo pathway through deubiquitinating the transcriptional coactivator Yorkie. Nat Commun. 2019;10:411. 30679505 10.1038/s41467-019-08334-7 PMC6345853

[43] Hu M, Li P, Li M, Li W, Yao T, Wu JW, et al. Crystal structure of a UBP-family deubiquitinating enzyme in isolation and in complex with ubiquitin aldehyde. Cell. 2002;111:1041–154. 12507430 10.1016/s0092-8674(02)01199-6

[44] Pozhidaeva A, Valles G, Wang F, Wu J, Sterner DE, Nguyen P, et al. USP7-specific inhibitors target and modify the enzyme’s active site via distinct chemical mechanisms. Cell Chem Biol. 2017;24:1501–1512.e1505. 29056420 10.1016/j.chembiol.2017.09.004

[45] Chauhan D, Tian Z, Nicholson B, Kumar KG, Zhou B, Carrasco R, et al. A small molecule inhibitor of ubiquitin-specific protease-7 induces apoptosis in multiple myeloma cells and overcomes bortezomib resistance. Cancer Cell. 2012;22:345–58. 22975377 10.1016/j.ccr.2012.08.007 PMC3478134

[46] Kessler BM. Selective and reversible inhibitors of ubiquitin-specific protease 7: a patent evaluation (WO2013030218). Expert Opin Ther Pat. 2014;24:597–602. 24456106 10.1517/13543776.2014.882320

[47] Barbieri CE, Baca SC, Lawrence MS, Demichelis F, Blattner M, Theurillat JP, et al. Exome sequencing identifies recurrent SPOP, FOXA1 and MED12 mutations in prostate cancer. Nat Genet. 2012;44:685–9. 22610119 10.1038/ng.2279 PMC3673022

[48] Le Gallo M, O’Hara AJ, Rudd ML, Urick ME, Hansen NF, O’Neil NJ, et al. Exome sequencing of serous endometrial tumors identifies recurrent somatic mutations in chromatin-remodeling and ubiquitin ligase complex genes. Nat Genet. 2012;44:1310–5. 23104009 10.1038/ng.2455 PMC3515204

[49] Cheng X, Xu X, Chen D, Zhao F, Wang W. Therapeutic potential of targeting the Wnt/β-catenin signaling pathway in colorectal cancer. Biomed Pharmacother. 2019;110:473–81. 30530050 10.1016/j.biopha.2018.11.082

[50] Morin PJ, Sparks AB, Korinek V, Barker N, Clevers H, Vogelstein B, et al. Activation of beta-catenin-Tcf signaling in colon cancer by mutations in beta-catenin or APC. Science. 1997;275:1787–90. 9065402 10.1126/science.275.5307.1787

[51] Kim JM, Song JS, Cho HH, Shin KK, Bae YC, Lee BJ, et al. Effect of the modulation of leucine zipper tumor suppressor 2 expression on proliferation of various cancer cells functions as a tumor suppressor. Mol Cell Biochem. 2011;346:125–36. 20890637 10.1007/s11010-010-0599-y

[52] Dong Z, Li J, Dai W, Yu D, Zhao Y, Liu S, et al. RRP15 deficiency induces ribosome stress to inhibit colorectal cancer proliferation and metastasis via LZTS2-mediated β-catenin suppression. Cell Death Dis. 2023;14:89. 36750557 10.1038/s41419-023-05578-6 PMC9905588

[53] Sun L, Xing J, Zhou X, Song X, Gao S. Wnt/β-catenin signalling, epithelial-mesenchymal transition and crosslink signalling in colorectal cancer cells. Biomed Pharmacother. 2024;175:116685. 38710151 10.1016/j.biopha.2024.116685

[54] Sudo H, Maru Y. LAPSER1/LZTS2: a pluripotent tumor suppressor linked to the inhibition of katanin-mediated microtubule severing. Hum Mol Genet. 2008;17:2524–40. 18490357 10.1093/hmg/ddn153

[55] Li G, Ci W, Karmakar S, Chen K, Dhar R, Fan Z, et al. SPOP promotes tumorigenesis by acting as a key regulatory hub in kidney cancer. Cancer Cell. 2014;25:455–68. 24656772 10.1016/j.ccr.2014.02.007 PMC4443692

[56] Bedford L, Lowe J, Dick LR, Mayer RJ, Brownell JE. Ubiquitin-like protein conjugation and the ubiquitin-proteasome system as drug targets. Nat Rev Drug Discov. 2011;10:29–46. 21151032 10.1038/nrd3321 PMC7097807

[57] Clevers H. Wnt breakers in colon cancer. Cancer Cell. 2004;5:5–6. 14749120 10.1016/s1535-6108(03)00339-8

[58] Nero TL, Morton CJ, Holien JK, Wielens J, Parker MW. Oncogenic protein interfaces: small molecules, big challenges. Nat Rev Cancer. 2014;14:248–62. 24622521 10.1038/nrc3690

[59] Zhou Z, Xu C, Chen P, Liu C, Pang S, Yao X, et al. Stability of HIB-Cul3 E3 ligase adaptor HIB Is Regulated by Self-degradation and Availability of Its Substrates. Sci Rep. 2015;5:12709. 26263855 10.1038/srep12709 PMC4533009

[60] Liu Q, He L, Li S, Li F, Deng G, Huang X, et al. HOMER3 facilitates growth factor-mediated β-Catenin tyrosine phosphorylation and activation to promote metastasis in triple negative breast cancer. J Hematol Oncol. 2021;14:6. 33407765 10.1186/s13045-020-01021-x PMC7788750

