# Supplementary material for: SPOP and HAUSP bidirectionally regulate LZTS2 ubiquitination to modulate the Wnt pathway
Source: Cell Death Dis. 2025 Dec 24;17(1):132. doi: 10.1038/s41419-025-08351-z (PMC12848046; doi:10.1038/s41419-025-08351-z)

**Supplementary information**

**SPOP and HAUSP bidirectionally regulate LZTS2 ubiquitination to modulate the Wnt pathway**


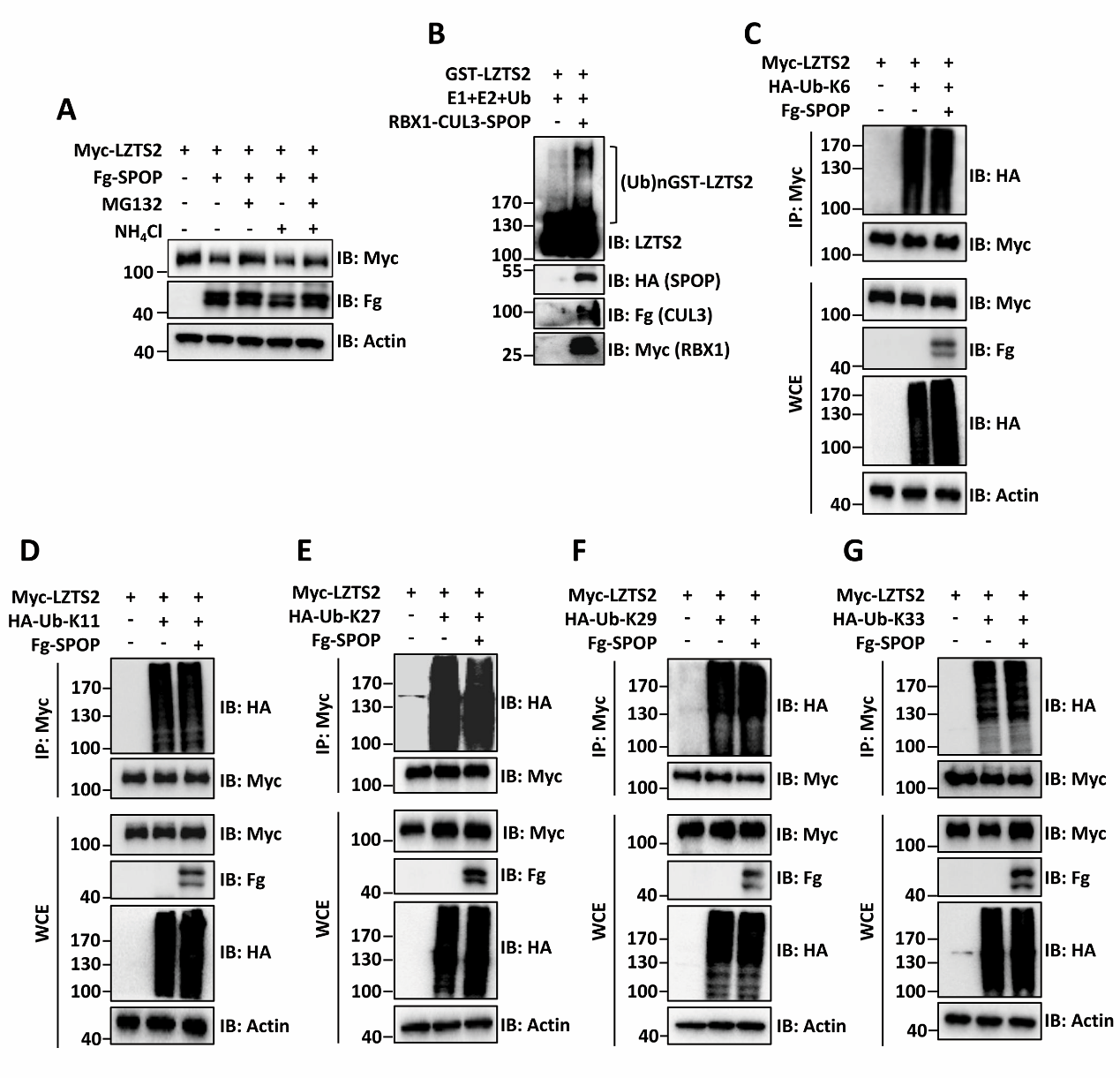
Yanran Deng et al.

**Figure S1. SPOP does not mediate K6-, K11-, K27-, K29-, or K33-linked ubiquitination of LZTS2.**

(**A**) Treatment of 293T cells with the proteasome inhibitor MG132 for 4 h, but not with the lysosome inhibitor NH_4_Cl, blocked the degradation of Myc-LZTS2 induced by Fg-SPOP. (**B**) *In vitro* ubiquitination assay showed that SPOP directly ubiquitinates LZTS2. (**C-G**) SPOP was unable to form K6- (C), K11- (D), K27- (E), K29- (F), or K33-linked (G) polyubiquitin chains on LZTS2.


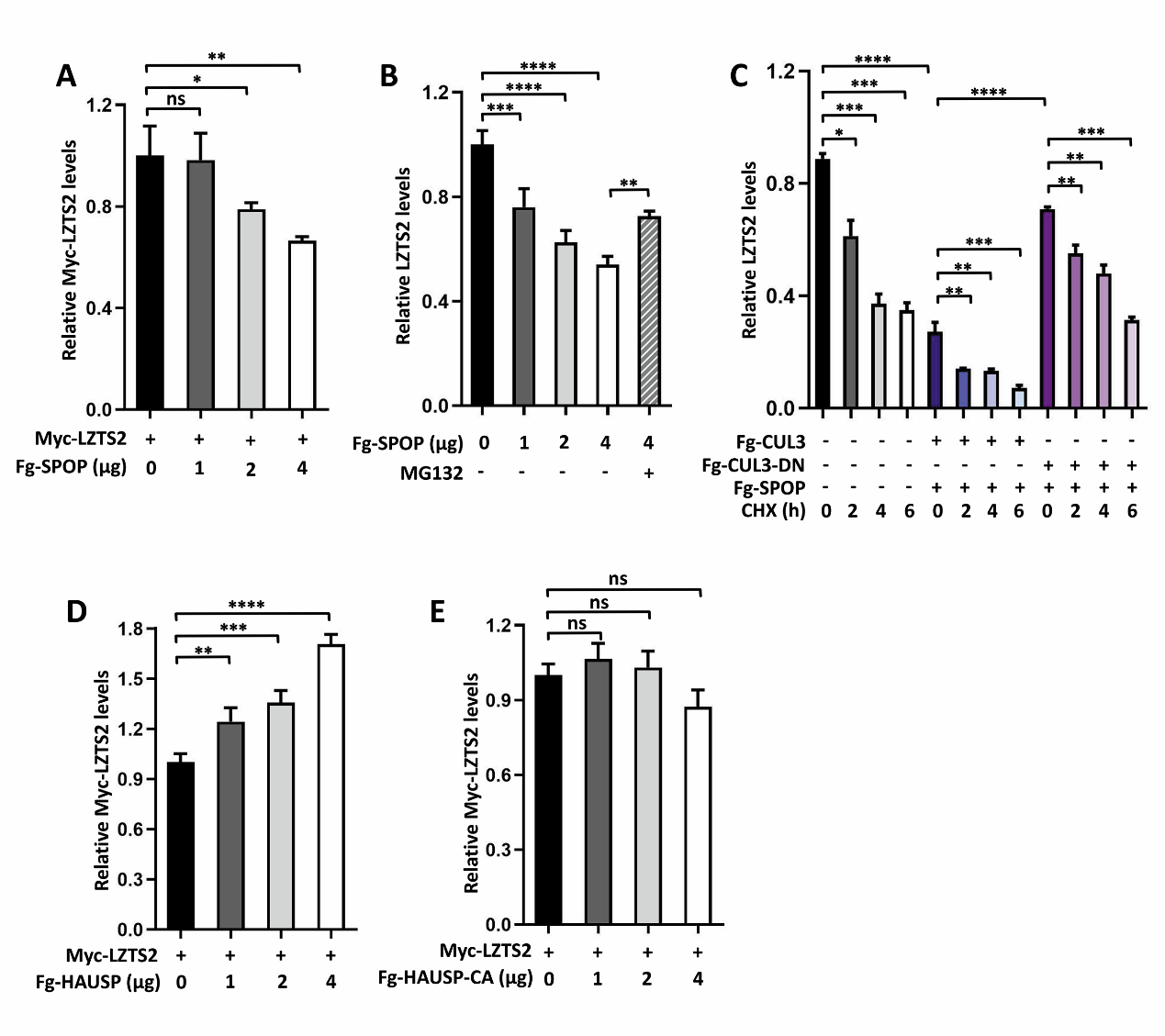
**Figure S2. SPOP degrades LZTS2 in a CUL3-dependent manner.**

(**A-E**) Quantitative analysis of LZTS2 or Myc-LZTS2 protein levels from the experiments shown in Figures 2A, 2B, 2C, 4A, and 4D, respectively.

**
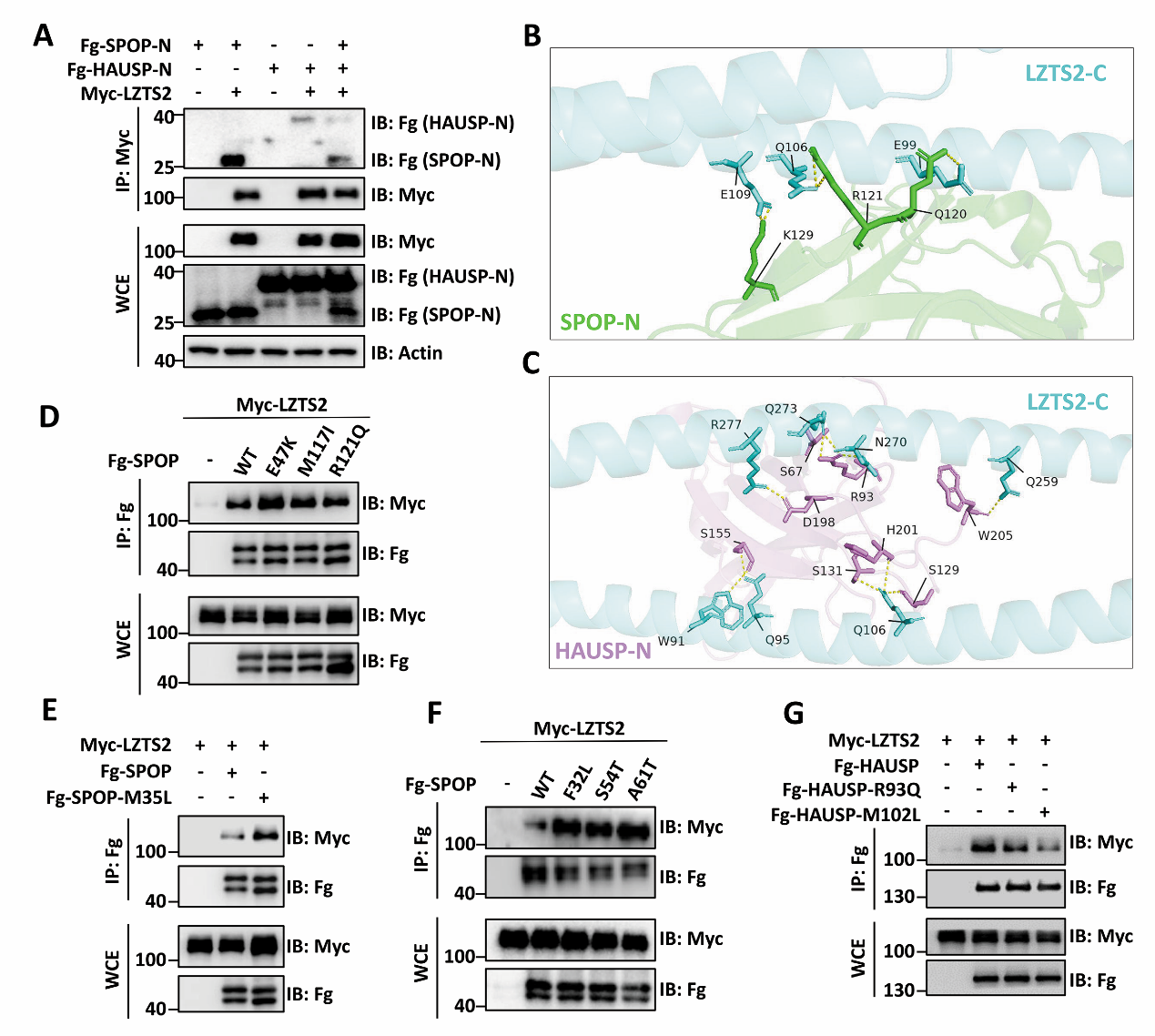
Figure S3. Cancer-derived mutations in SPOP and HAUSP modulate their affinity with LZTS2.**

(**A**) HAUSP-N and SPOP-N competed with each other for binding to LZTS2. (**B**) AlphaFold model of the SPOP-N (green) in complex with LZTS2-C (blue). (**C**) AlphaFold model of the HAUSP-N (purple) in complex with LZTS2-C (blue). (**D**) 293T cells were transfected with the indicated plasmids and treated with 25μM of MG132 for 4 h before cell harvesting. Endometrial cancer-associated SPOP mutants exhibited comparable interaction with LZTS2 as wild-type SPOP. (**E**) Hepatocellular carcinoma-derived SPOP mutant, SPOP-M35L, showed an increased binding affinity towards LZTS2. (**F**) Colorectal cancer-associated HAUSP mutants exhibited reduced affinity to Myc-LZTS2. (**G**) Colorectal cancer-associated SPOP mutants showed increased affinity to Myc-LZTS2.

**
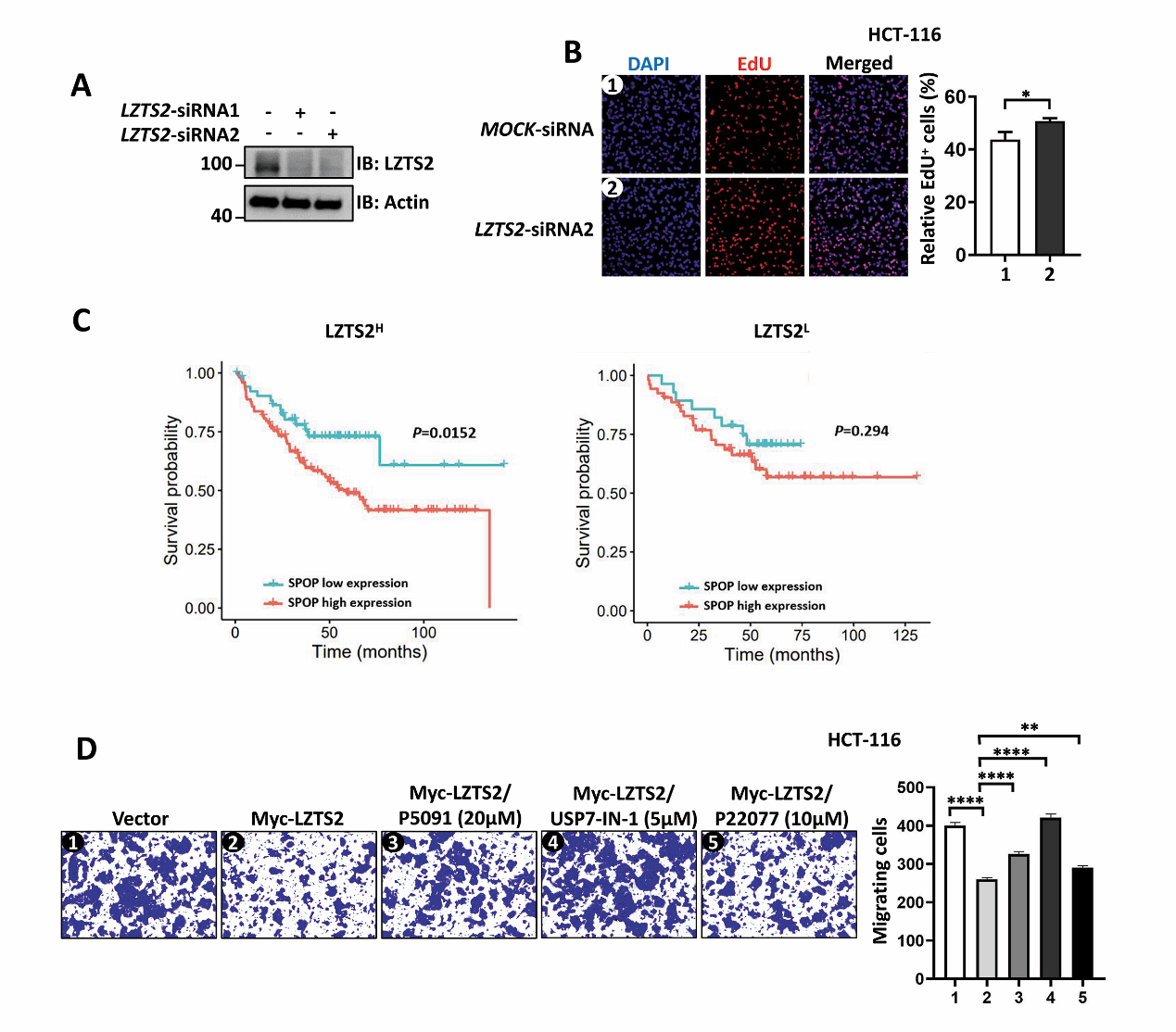
Figure S4. Knockdown of LZTS2 promotes CRC cell proliferation.**

(**A**) Immunoblots of whole cell lysates from HCT-116 cell transfected with indicated

siRNAs. Actin acts as a loading control. (**B**) Knockdown of LZTS2 increased HCT-116 cell proliferation. Quantitative analysis was shown on the right. (**C**) Kaplan-Meier analysis comparing overall survival between CRC patients with low and high SPOP expression in the LZTS2^H^ and LZTS2^L^ cohorts. Curve comparison was performed with log-rank (Mantel-Cox) test. (**D**) Treatment with HAUSP inhibitors reduced LZTS2-mediated HCT-116 cell migration inhibition.


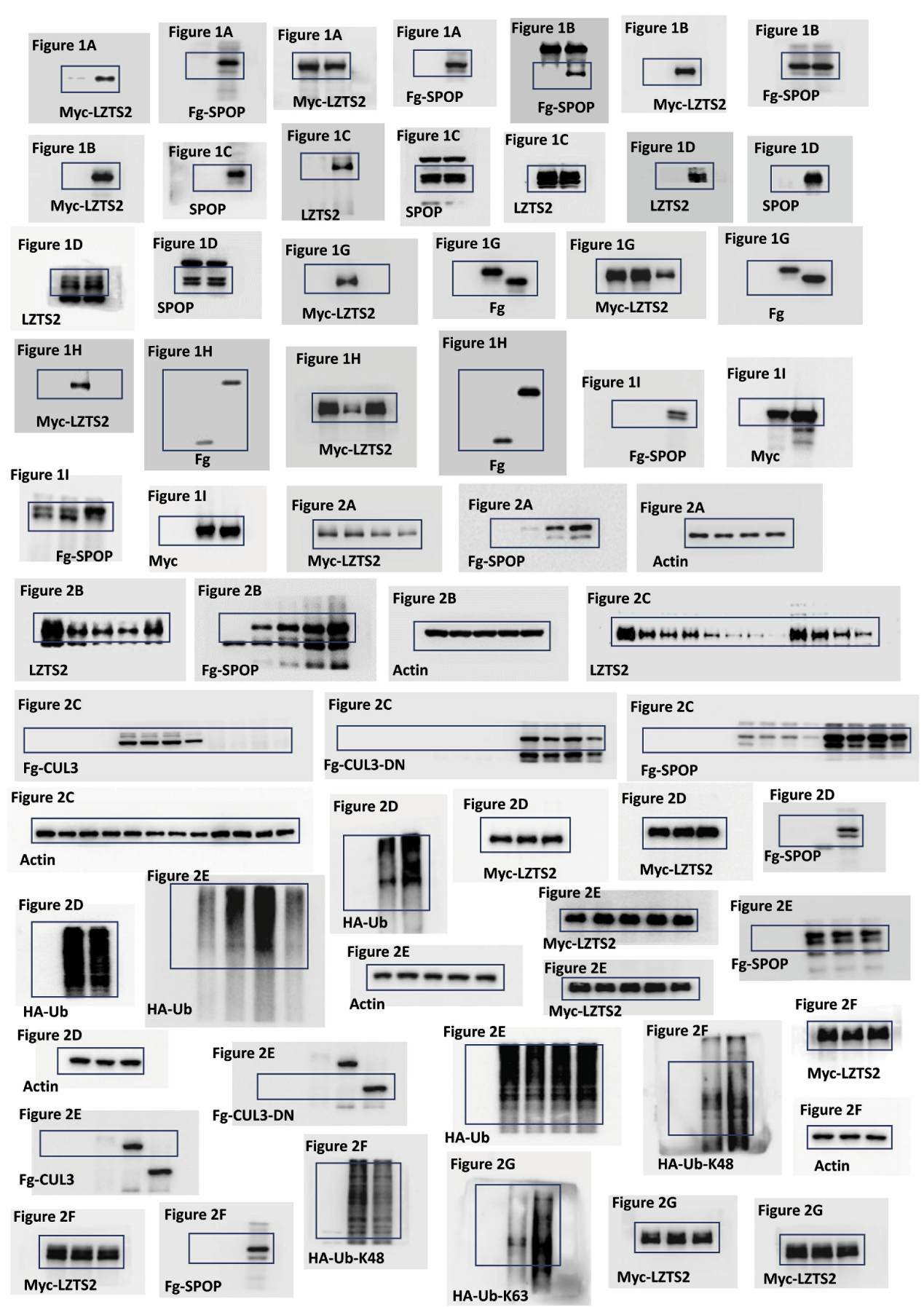
Original WB data:


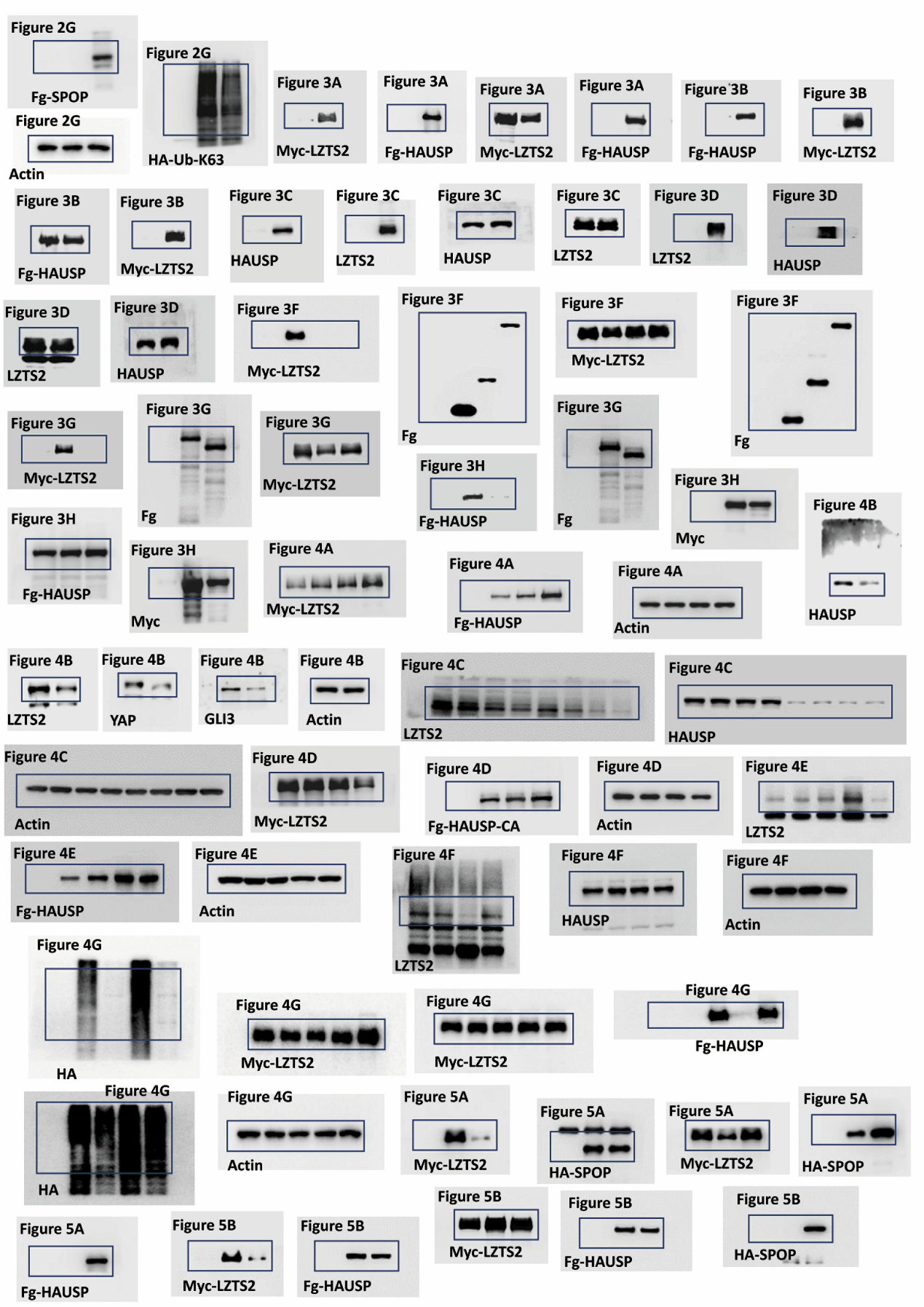


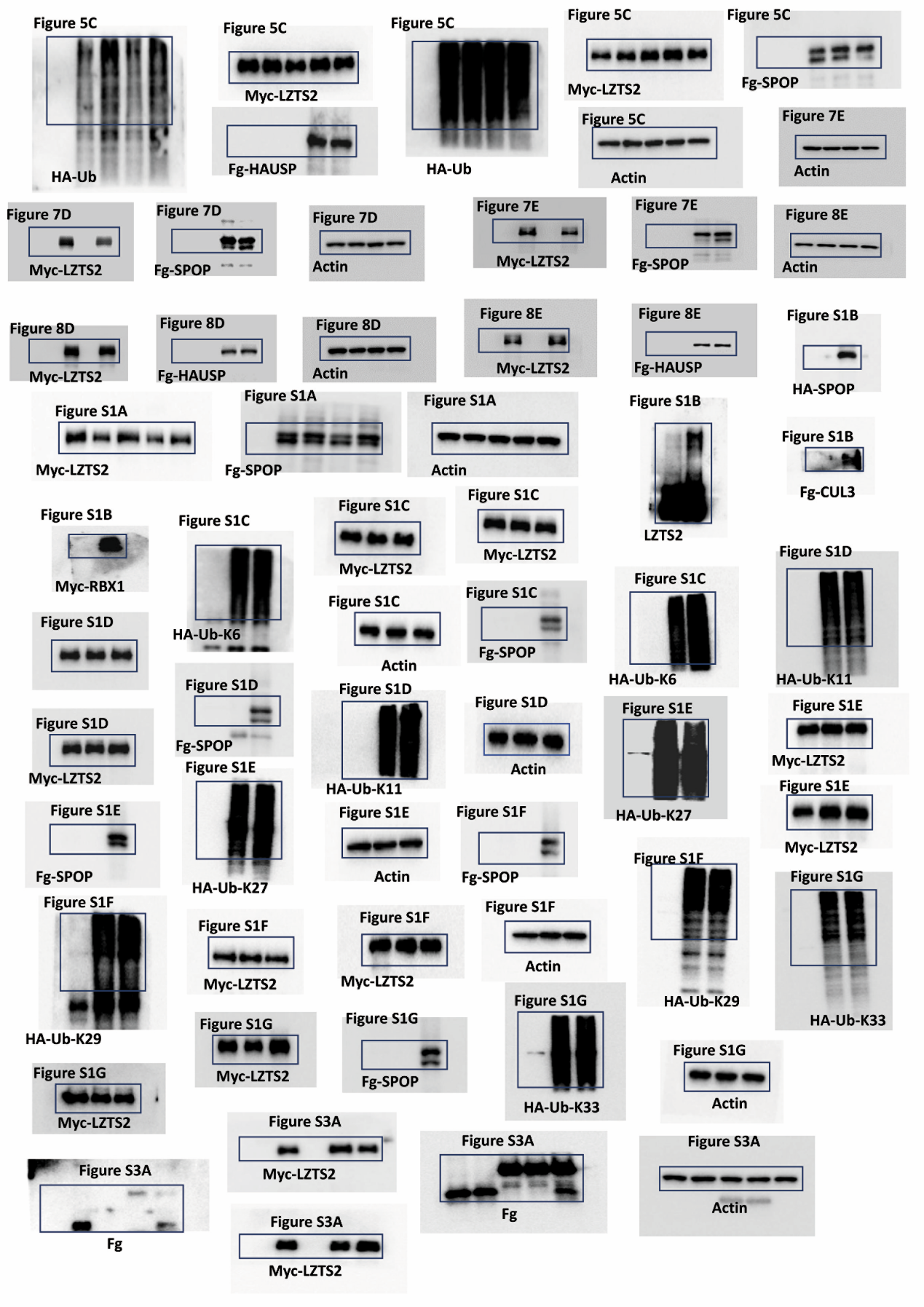


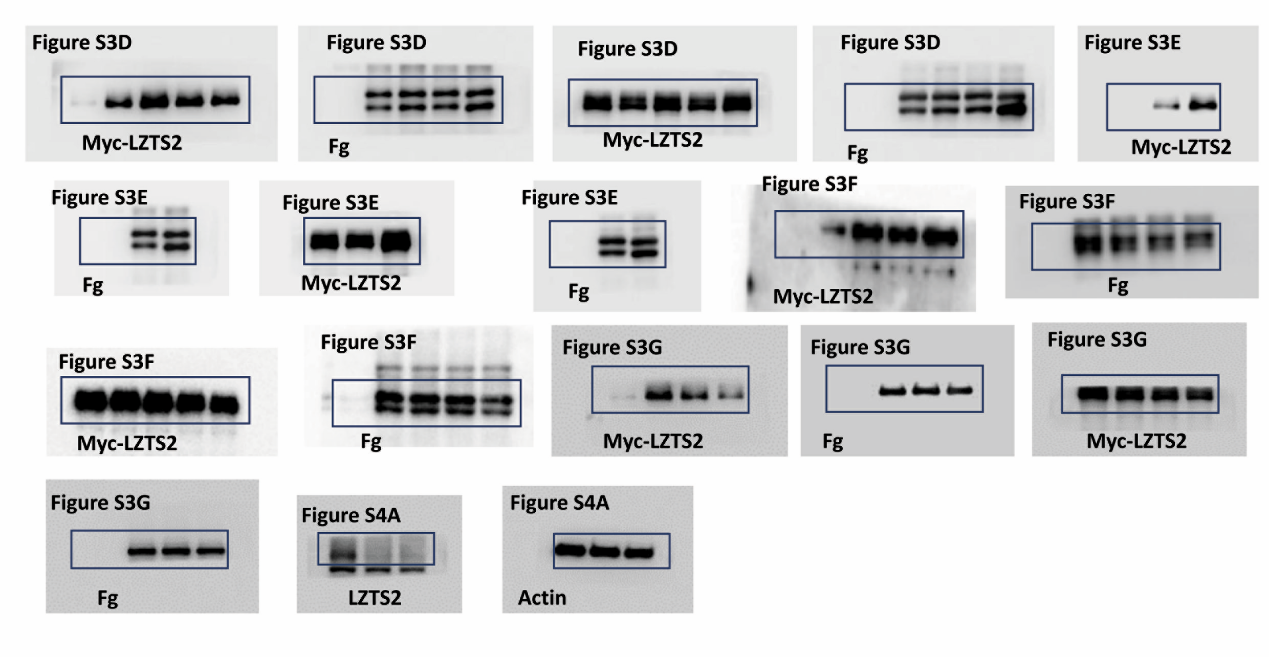

Supplement: Supplementary file 1 — Supplementary Information [file 41419_2025_8351_MOESM1_ESM.docx]
